# How exposure to ALS-inhibiting gametocide tribenuron-methyl induces male sterility in rapeseed

**DOI:** 10.1186/s12870-019-1722-1

**Published:** 2019-04-02

**Authors:** Jing-long Lian, Li-Suo Ren, Cong Zhang, Cheng-Yu Yu, Zhen Huang, Ai-Xia Xu, Jun-Gang Dong

**Affiliations:** College of Agronomy, Northwest A&F University, Yangling, 712100 Shaanxi China

**Keywords:** Rapeseed, Male sterility, Gametocide, Acetolactate synthase, Tribenuron, Plastid

## Abstract

**Background:**

Acetolactate synthase (ALS)-inhibiting herbicide tribenuron-methyl (TBM) is an efficient gametocide that can cause rapeseed (*Brassica napus* L.) to become male sterile and outcrossing. To find the reason the TBM treatment leads to male sterility, an integrated study using cytological, physiological, and transcriptomic methods was conducted.

**Results:**

Some temporary symptoms, including the discoloration of young leaves and a short halt of raceme elongation, were observed in the rapeseed plants exposed to TBM at an application rate of 1 μg per plant. Both chloroplasts in young leaves and plastids in anthers were deformed. TBM also reduced the leaf photosynthetic rate and the contents of chlorophyll, soluble sugar and pyruvate. Both the tapetal cells and uni-nucleate microspores in the treated plants showed large autophagic vacuoles, and the tissue degenerated quickly. A transcriptomic comparison with the control identified 200 upregulated and 163 downregulated differential expression genes in the small flower buds of the TBM treatment. The genes encoding functionally important proteins, including glucan endo-1,3-beta-glucosidase A6, QUARTET3 (QRT3), ARABIDOPSIS ANTHER 7 (ATA7), non-specific lipid-transfer protein LTP11 and LTP12, histone-lysine N-methyltransferase ATXR6, spermidine coumaroyl-CoA acyltransferase (SCT), and photosystem II reaction centre protein psbB, were downregulated by TBM exposure. Some important genes encoding autophagy-related protein ATG8a and metabolic detoxification related proteins, including DTX1, DTX6, DTX35, cytosolic sulfotransferase SOT12, and six members of glutathione S-transferase, were upregulated. In addition, several genes related to hormone stimulus, such as *1-aminocyclopropane-1-carboxylate synthase 8* (*ACS8*), ethylene-responsive factor *ERF1A, ERF1, ERF71, CRF6,* and *RAP2-3*, were also upregulated. The transcriptional regulation is in accordance with the functional abnormalities of pollen wall formation, lipid metabolism, chloroplast structure, ethylene generation, cell cycle, and tissue autophagy.

**Conclusion:**

The results suggested that except for ALS, the metabolic pathways related to lipid metabolism, pollen exine formation, photosynthesis and hormone response are associated with male sterility induced by TBM. The results provide new insight into the molecular mechanisms of inducing male sterility by sulfonylurea.

**Electronic supplementary material:**

The online version of this article (10.1186/s12870-019-1722-1) contains supplementary material, which is available to authorized users.

## Background

Acetolactate synthase (ALS; EC 4.1.3.18) is a key enzyme in the biosynthesis of leucine, valine, and isoleucine, which are branched-chain amino acids (BCAAs) and can be inhibited by various herbicides of group B (according to the Herbicide Resistance Action Committee classification) from the sulfonylurea (SU), imidazolinone, triazolopyrimidine, pyrimidinylbenzoate, and sulfonylamino-carbonyltriazolinone families [1]. Though ALS is known to be the common target of dozens of herbicides, the reason why ALS-inhibitors cause phytotoxicity remains highly elusive. Various hypotheses, including the depletion of the BCAA pool, blocking of DNA synthesis, accumulation of substrates, and blocking of photoassimilate translocation, as well as anaerobic respiration, have been suggested as the mechanism of the cytotoxicity of ALS-inhibiting herbicides based on physiological traits, cellular alterations, and the metabolic investigation of the plants and microbes exposed to ALS-inhibiting herbicides (reviewed by Zhou et al., [2]). Unfortunately, there is still not a consensus on the mechanism for cell death caused by ALS-inhibiting herbicides.

Except for the herbicidal activity of ALS-inhibitors, many members of SU and the imidazolinone family, for example, tribenuron-methyl (TBM), amidosulfuron, and imazethapyr (IM), were identified to be good gametocides that can induce complete male sterility (MS) in many cruciferous species or some cereal plants when applied at sub-lethal amounts [3–10]. This MS belongs to chemically induced MS (CIMS). In plant breeding, both inheritable MS (genic male sterility and cytoplasmic male sterility) and CIMS are used to promote outcrossing in the maternal line of hybrid seed production. The main advantages of the CIMS method over inheritable MS is that most inbred lines or cultivars can be used as the parent lines of hybrids, and different lines need not be created for an MS system, i.e., male sterile line, maintainer, and restorer. In addition, many heritable MS systems showed poor seed-set, quality traits, and/or disease resistance of their hybrids [11–13]. By now, breeding based on CIMS has had great success in China, and more than 20 commercial hybrid rapeseed (*Brassica napus* L.) varieties based on CIMS have been registered [10].

Several institutes have conducted transcriptomic analyses to determine the reason for the phytotoxicity of ALS-inhibiting herbicides. Manabe et al., [14] identified some genes of defence and detoxification at the early stage after IM application and other genes involved in the biosynthesis of amino acids and secondary metabolites at a later stage by a comparison between IM-sensitive and resistant *Arabidopsis thaliana* mutants. The differences between the SU herbicide treatment of primisulfuron and prosulfuron can be showed by DNA array detection using *A. thaliana* genes that belong to the secondary metabolism [15]. Similarly, the transcriptional changes of a few genes could differentiate the responses of *A. thaliana* and *B. napus* to several closely related herbicides [16]. The mechanism of MS occurring in the plants susceptible to a sub-lethal rate (approximately 1 to 5% of the dose recommended for weed control) of these herbicides remains unknown. A few other studies [8–10] investigated the mechanism of CIMS by ALS-inhibiting gametocides, such as monosulfuron ester sodium (MES, which belongs to the SU family), imazethapyr (IM, belongs to the imidazolinone family), and amidosulfuron. The blocking of carbohydrate and lipid metabolism, the destruction of chloroplasts and autophagic cell death were suggested in these studies [7–10, 17, 18].

The MS induced by ALS-inhibiting herbicides give us a good chance to study the phytotoxic effect of these herbicides, especially at a sub-lethal dose. Although some SUs and imidazolinones can elicit CIMS in Brassica or other plants [3–10], the inhibition of the ALS enzyme is not a guaranty of CIMS. Some triazolopyrimidine and pyrimidinylthiobenzoate herbicides cannot cause MS, though they also inhibited the activity of the ALS enzyme [5]. It seems that some other biological pathways are also necessary for ALS-inhibition gametocides to induce MS. The aim of this study was to investigate the cytological, physiological, and transcriptional changes of the rapeseed response to gametocide TBM exposure. The possible associations of these biological responses with MS were discussed. These results would be useful to better understand the mechanisms inducing MS by TBM and other ALS-inhibitors.

## Methods

### Plant material and TBM treatment

The plants of rapeseed cv. SP2F (TBM susceptible) were grown in the experimental field of Northwest A&F University (Yangling, Shaanxi, China), with a plant density of 15 seedlings per square metre. The bolting plants (fifteen days before flower opening) were foliar-sprayed by a working solution containing of 0.2 mg/L (available ingredient) TBM (Express™) and 0.2 mL/L surfactant sodium alkylethersulfate, at a rate of approximately 5 mL per plant (the calculated dose of TBM is 1 μg per plant, equal to 150 mg/ha). A handheld high pressure pump sprayer was used to evenly moisten the leaves. This treatment had three replicates, with each plot containing approximately 100 plants. Another group of plants, which served as the negative control, were sprayed with only water containing the surfactant. An *als* mutant DS3 (genotype TBM-R) that was donated by the Institute of Industrial Crop, Jiangsu Academy of Agriculture Science [19] was also treated with the TBM solution to compare the genotypic effect.

### Cytological observations

An aceto-carmine staining of anthers was performed to examine the microspore and pollen developmental stage [10]. Leaves and flower buds from the TBM-treated plants and control were collected according to their developmental stages. The process of section preparation was the same as in our previous study [10].

### Assay of physiological traits

Since ALS is the main target enzyme of TBM, the in vivo activities of ALS enzymes in mature leaves and young flower buds (length ≤ 3 mm) were assayed several days after treatment (DAT), as in our previous study [10]. Student’s *t*-test was used to simultaneously compare the same collected tissues for each of three replicates. The in vivo ALS activity, which was decided by accumulation of acetolactate in the tissue, is often used to determine the ALS activity inhibition by the herbicide that is absorbed and/or translocated by plant. This method also concerns the substrate supply or metabolic detoxification of the herbicide. Moreover, to find biological pathways other than ALS that are affected by TBM, more physiological traits were determined, especially those related to the photosynthesis system, owing to leaf discoloration by TBM. The photosynthetic rate of the upper mature leaves of five plants in each treatment was measured 0, 2, 4, 6 DAT using our previous method [10]. The contents of chlorophyll, pyruvate acid, and soluble sugar in the leaves and the ethylene release rate in the flower buds were assayed as in our previous study [10].

### Transcriptomic analysis

The young flower buds (length ≤ 3 mm) corresponding to the key stages sensitive to gametocides, that is, the microsporocyte (or microspore mother cell, MMC) to uni-nucleate microspore stages, were dissected from the plants 5 DAT, as well as from the control plants. The two biological replicates were designed as HS1 and HS2 for the treatment and CK1 and CK2 for the control. The total RNA was extracted and reverse transcribed to cDNA using our previous method [10]. Two groups of Illumina DGE (digital gene-expression tag profiling) libraries were constructed using the aforementioned cDNAs of young flower buds. We performed the single end sequencing on an Illumina Hiseq2500 platform following the vendor’s protocol. All reads were deposited in NCBI under the Gene Expression Omnibus accession number GSE113681. After a quality check and data filter [10], the obtained clean reads with a length of 36 nt were aligned with *Brassica* Gene Index Databases (Dana-Farber Cancer Institute (DFCI), Boston, MA 02115, USA. URL: http://compbio.dfci.harvard.edu/tgi or ftp://occams.dfci.harvard.edu/pub/bio/tgi/data/) by using Bowtie v2.1.0 (http://sourceforge.net/projects/bowtie-bio/files/bowtie2/2.1.0/). The number of perfect reads matching each unigene was normalized to transcripts per kilobase of exon model per million mapped reads (TPM). The low-frequency transcripts were filtered, and the significant DETs among the two groups of samples were selected [10]. The sequence of each DET was further searched by BLAST to get more information from the NCBI nucleotide collection database. The functional annotation was searched in the UniProt database (http://www.uniprot.org/). Some selected DETs were clustered by using Cluster 3.0 (http://bonsai.hgc.jp/~mdehoon/software/cluster/software.htm), according to the relative expression values transformed from their TPM. The genetic networks of GO (Gene Ontology) terms for the DETs were predicted by the BiNGO software in Cytoscape (www.cytoscape.org/). All possible protein-protein interactions among the DETs were further revealed by the STRING platform (https://string-db.org/).

### Quantitative real-time PCR (qPCR) for gene expression analysis

The procedure of qPCR is similar to our previous study with minor modifications [10]. The RNA was extracted by using HiPure Plant RNA Mini Kit (Magen, Guangzhou, China). Two *ALS* loci (*ALS1* and *ALS3*) encoding the proteins with necessary function for *B. napus* [17] and other 16 selected DETs were tested by qPCR using specific primers (Additional file 1: Table S1) that were designed according to corresponding cDNA sequences. PCR assays were performed on a QuantStudio 3 thermal cycler (Thermo Fisher Scientific, CA, USA).

## Results

### Morphological changes of rapeseed exposed to a sub-lethal dose of TBM

**Fig. 1:**
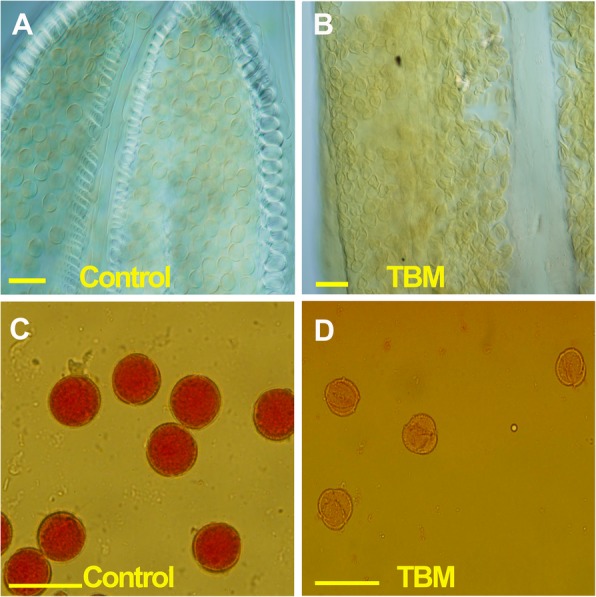
Comparison of the pollen grains between the control and treated plants. **a** Pollen grains are round in shape in the control but malformed in the TBM-treatment (**b**). **c** Pollen grains are deep stained by the aceto-carmine in the control, while they are not stained under TBM treatment (**d**).

**Fig. 2:**
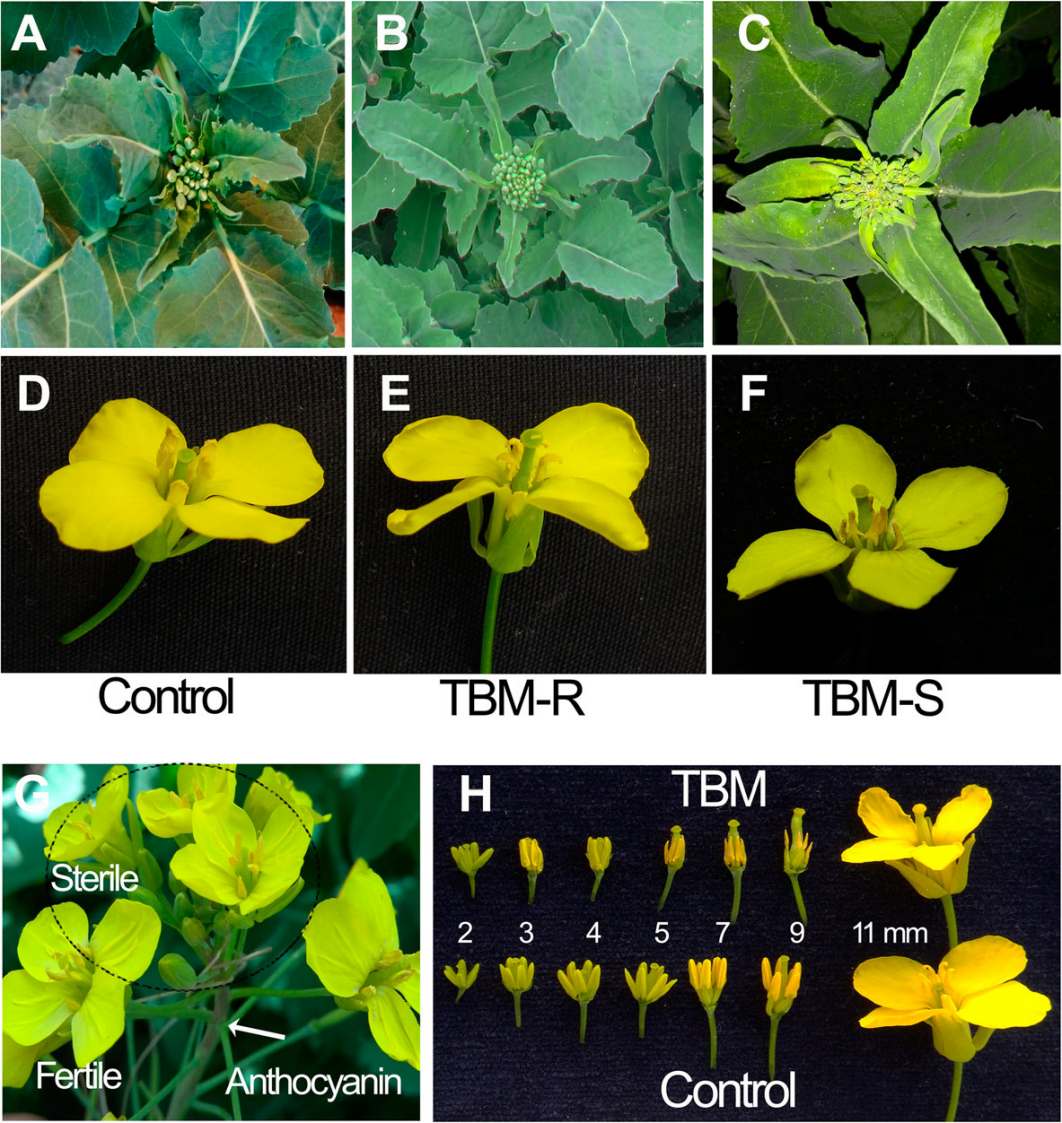
Morphological characteristics and pollen grains of TBM-treated plants. **a** Leaves of control. **b** Leaves of TBM-R after TBM treatment. **c** Leaf discoloration on the TBM-treated plant. The corresponding flowers are shown in (**d**), (**e**), and (**f**) in the bottom panel. **g** Inflorescence transferring from male fertile to sterile phase. Arrowhead indicates anthocyanin deposition. **h** Size reduction of filament and anther in the TBM-treated flower buds ≤3 mm

The anthers of the TBM-treated plants were shrivelled and contained only dead pollen grains that were deformed and not stained by aceto-carmine (Fig. 1). In addition to the MS phenotype, TBM had some morphological influences on rapeseed, including the temporary depression of the stem elongation and discoloration of young leaves in the first several days, indicating a phytotoxic effect on cell growth, chloroplast structure, and/or flavonoid biosynthesis. The exposure to 5 mL of 0.2 mg/L TBM per plant resulted in no reduction in mature plant height but did cause a 1-2 day delay in the flowering time and a short retardation of raceme elongation. To offset the metabolic detoxification during the long period of flowering that can last for three weeks, TBM exposure of a higher dose was used to prolong the gametocidal effect on the anther. Therefore, some side effects, such as discoloration in the upper leaf (Fig. 2c) and the deposition of anthocyanin (Fig. 2g) one week after treatment, were observed in the treated plants. The TBM-resistant genotype TBM-R did not show these symptoms, nor did the MS phenotype under the dose treatment (Fig. 2b).

### Cytological observation of MS induced by TBM

**Fig. 3:**
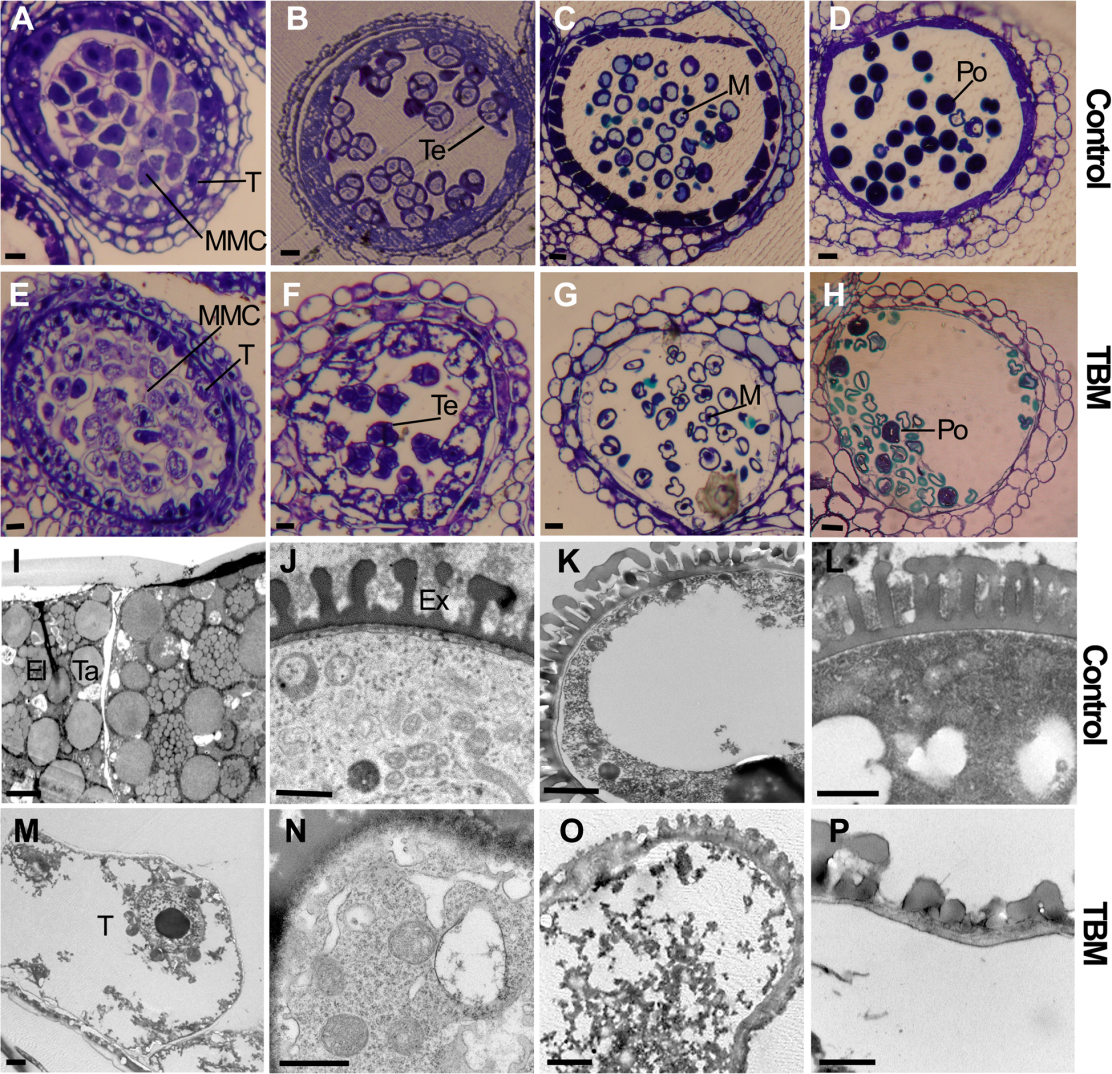
Structure of anthers and pollen of TBM-treated plants*.* (A-H are stained with toluidine blue and the scale bar = 20 μm; I-P are transmission electron microscope images with scale bar = 1 μm.). **a** Normal microsporocyte mother cell. **b** Normal tetrad. **c** Uni-nucleate microspore. **d** Near-mature pollen with deep stain. **e** TBM-treated microsporocyte mother cells and tapetal cells are loosely arranged. **f** Malformed tetrad microspores. **g** TBM-treat tapetal cells disappear at the uni-nucleate stage. **h** Several pollen grains can be stained by toluidine blue, but most unviable pollen grains are deformed. **i** Normal tapetum full of elaioplasts and tapetosomes at the late uni-nucleate microspore stage. **j** Early microspore with exine. **k** Late uni-nucleate microspore. **l** Mature pollen with well-formed exine. **m** Tapetum is degraded at the uni-nucleate microspore stage. **n** Early microspore full of autophagic vacuoles. **o** Malformed microspore with undeveloped exine. **p** Unviable microspore loses cytoplasm, with undeveloped exine. Abbreviation: El, elaioplast; Ex, exine; M, microspore; MMC, microspore mother cell; Po, pollen grain. T, tapetum; Ta, tapetosome; Te, tetrad

Some obvious defects were observed in either the tapetal cells, the microsporocyte or the microspore at all stages of the developmental process of the microspore and pollen in TBM-treated plants but were not observed in the control (Fig. 3a-d). A normal tapetal cell and microspore contain abundant organelles (Fig. 3a-d). However, during the stage of MMC meiosis, tetrad-formation, and microspore release from the tetrads, the tapetal cells of the TBM treatment had much larger vacuoles than the control (Fig. 3e, f). After the enlargement of the tapetal cells and the expansion of the vacuoles, tapetal cells started to be degraded (Fig. 3f). Tetrad microspores were irregularly shaped compared with the control (Fig. 3f). Both MMCs and tapetal cells showed more space left by the shrivelled nucleus and cytoplasm (Fig. 3e, f). Then, at the uni-nucleate microspore stage, the tapetum and uni-nucleate microspore quickly broke down, with large vacuoles or cavities having formed in the microspore of the treated plants (Fig. 3g). The microspores could not become round (Fig. 3g, h). With the loss of the tapetum, the microspores were eventually degraded (Fig. 3h), and the anther locules became empty, apart from a cluster of microspore remains.

TEM observation showed more visible defects in the TBM treatment. Here, some obvious abnormalities were described. At the middle of the microspore stage, normal tapetal cells were congested by numerous elaioplasts and tapetosomes (Fig. 3i), but the structure of the tapetum in TBM-treated plants was very obscure in the structure, without such organelles as elaioplasts and tapetosomes (Fig. 3m) observed. At the uni-nucleate microspore stage, the tapetal cells started to degenerate, and they became less densely stained. (Fig. 3m). In the TBM-treated plants, microspore cytoplasm was degraded by many autophagic vacuoles, producing irregular and hollow cavities (Fig. 3n). Microspore wall development did not proceed, and the exine showed much poorer structural differentiation than the control (Fig. 3o, p). These results show the aberrant lipid metabolism and transportation in TBM-treated plants.

**Fig. 4:**
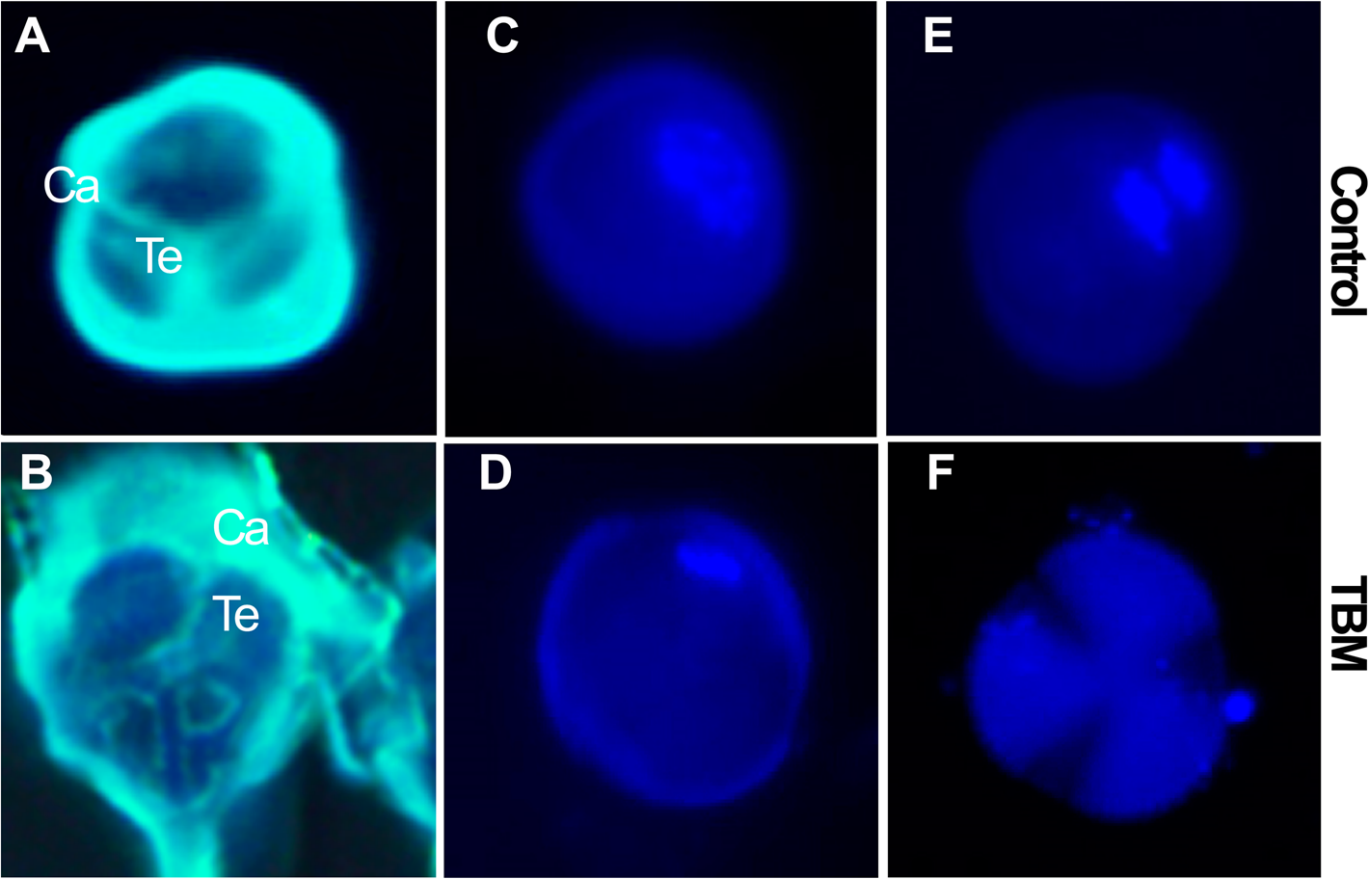
Abnormal callose layer covering the tetrads and stopped microspore development before mitosis in TBM-treated plants. A and B are stained by aniline blue and C to F are stained by DAPI. **a** Callose layer covering the microsporocytes of the control. **b** Deformed callose layer surrounding the tetrad microspores in TBM-treated plants. **c** The uni-nucleate microspore of the control. **d** The uni-nucleate microspore of TBM-treated plants contains a weak DAPI signal. **e** The bi-nucleate pollen grain after first mitosis contains abundant nuclear material with a strong DAPI signal. **f** The nuclear materials of the TBM-treated microspore disappear at this time, and the microspores do not undergo mitosis. Abbreviations: Ca, callose; Te, tetrad. Each pair of figures had the same scale bars of 10 μm

Further cytological observation found that during meiosis, MMC divided to form a tetrad encapsulated by a thick callose layer. However, the tetrad microspores and the callose layer were malformed (Fig. 4b), indicating abnormality in the cell structure and callose deposition and/or dissolution. In addition, at the late stage of the uni-nucleate microspore, the normal plants became bi-nucleate pollen, and the nucleus of TBM-treated plants gradually disappeared and could not fulfil microspore mitosis by the end of uni-nucleate microspore stage (Fig. 4d, f), indicating that TBM had a greater early effect before microspore mitosis. To confirm the key stage at which TBM works, we postponed TBM spraying on the normal rapeseed plants when the flowers begin to open. The flower buds of different sizes on the main raceme were indicated by thread or colour pen. The flower buds longer than 3.5 mm on the treated plants would maintain the fertile phase, and after one week, the subsequent flowers, whose initial sizes were 3 mm long and whose anther development was at the stage of late microspore when they were exposed to TBM, were converted to male sterile state. This observation also suggested that TBM restrains the microsporogenesis process before microspore mitosis, as amidosulfuron did [10].

### Effect of TBM treatment on chloroplast structure, photosynthesis and energy metabolism

**Fig. 5:**
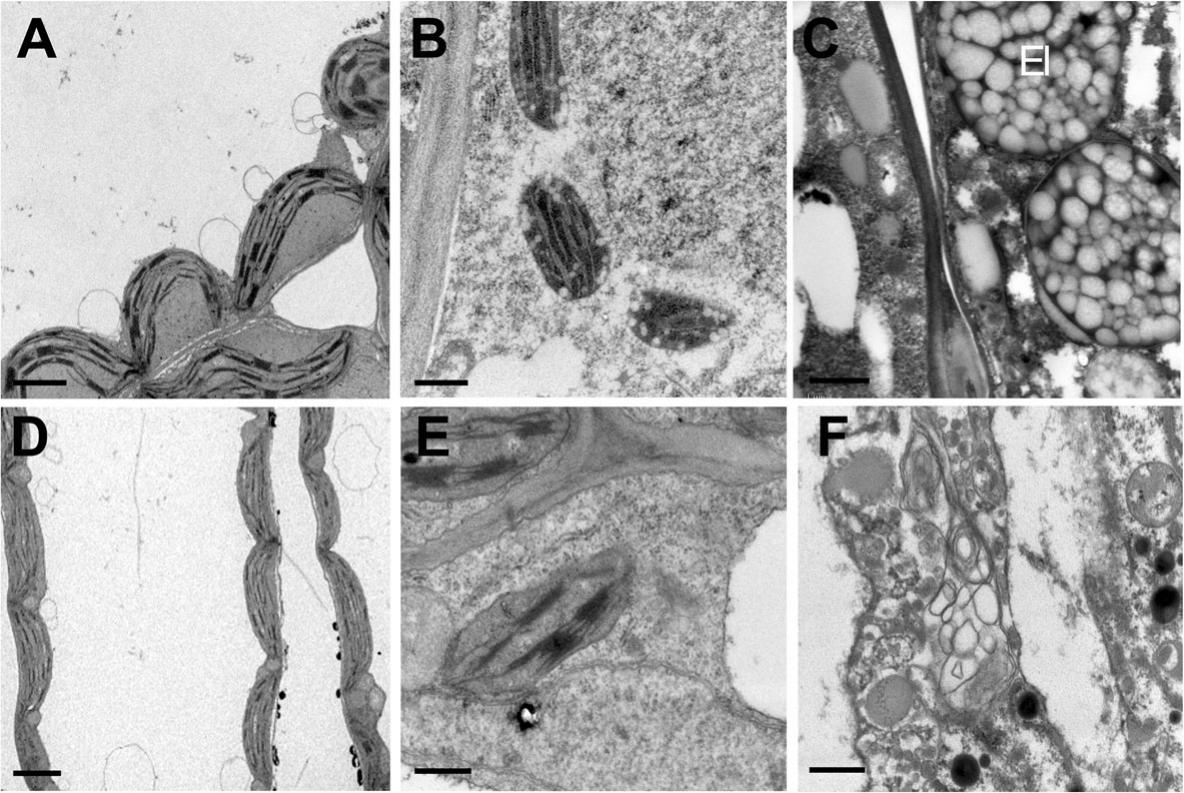
Comparison of chloroplasts and tapetal plastids between the control and treated plants. **a** The mesophyll cell chloroplasts of the control contain grana and thylakoid membranes. **b** Chloroplasts in the anther epidermis of the control contain thylakoid membranes and starch granules. **c** The magnified structures of the tapetum show normal elaioplasts with plastoglobuli accumulated. **d** The chloroplasts in the treated plants are flat and undeveloped. **e** Abnormal chloroplasts in the anther epidermis of the treated plants without grana stacks. **f** The malformed structure and defective organelles, including plastids, in the TBM-treated tapetum. Abbreviation: El, elaioplast. Scale bar = 2 μm

To know whether the symptom of leaf discoloration could affect the photosynthetic capability, we detected the plastid and chloroplast structure in the anthers and leaves. The normal chloroplasts were inflated, full of grana and thylakoid membranes (Fig. 5a, b). In contrast, the TBM-treated chloroplasts were undeveloped, and its grana were thin (Fig. 5d). These results suggest that TBM-treated plants may have a decreased photosynthetic rate. The chloroplasts in epidermal cells of the TBM-treated plants lost the thylakoid membrane and grana stacks (Fig. 5e). Tapetal elaioplasts were also destroyed by TBM exposure (Fig. 5f), and tapetosomes seldom formed, intimating an abnormality in pollen coat components such as sporopollenin produced by these tapetal organelles.

**Fig. 6:**
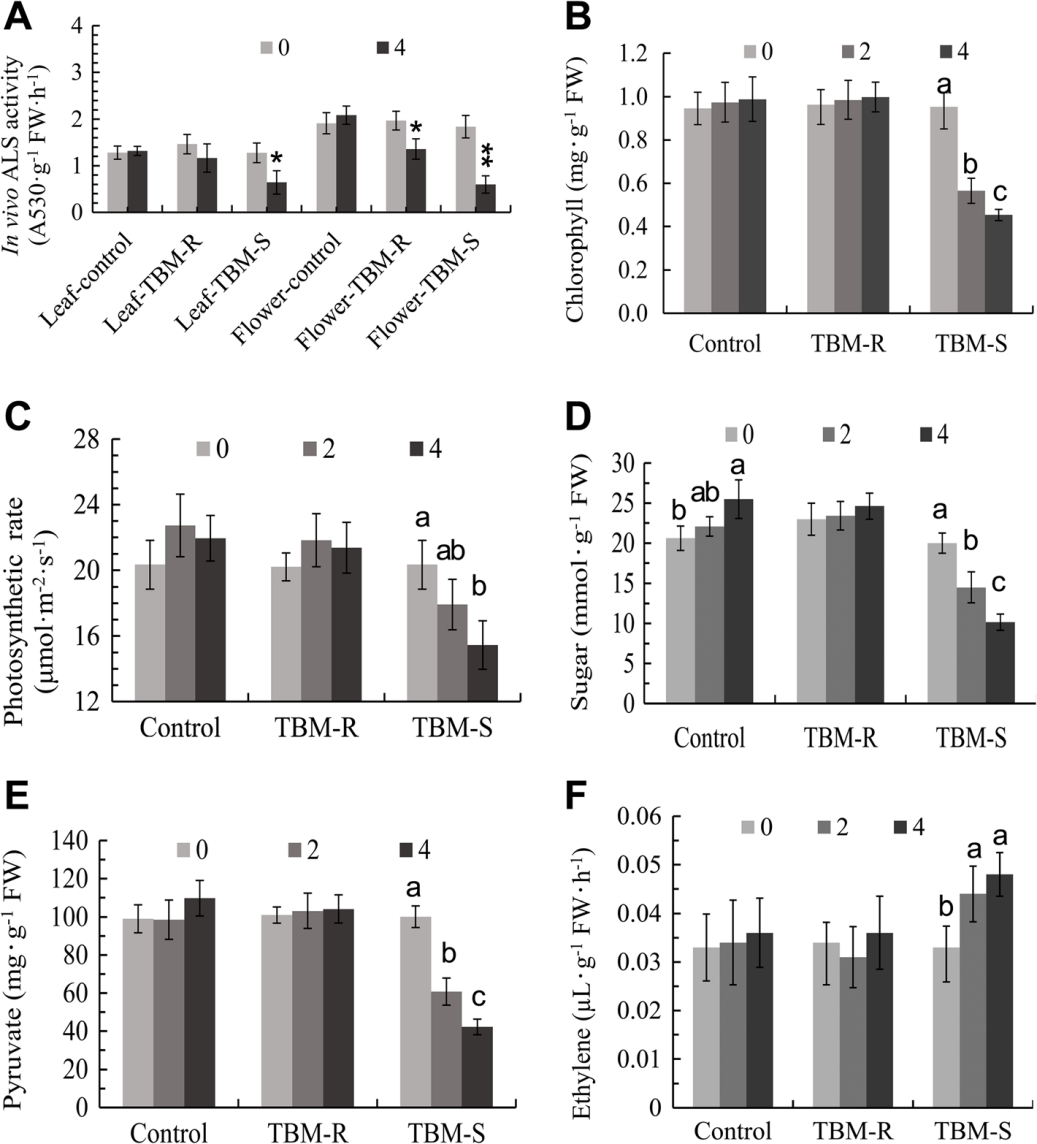
Effect of TBM on in vivo ALS activity, photosynthetic rate, the contents of leaf chlorophyll, soluble sugar, and pyruvate, and ethylene release rate in the flower buds 0, 2, and 4 days after treatment (DAT). Error bars represent the standard deviation. Note: the symbols * and ** indicate significant differences at 95 and 99% confidence levels by Student’s *t*-test, and the letters a, b, c indicate significant differences at 95%

The 0.2 mg/L TBM exposure obviously inhibited the in vivo ALS activity in both leaves and flower buds (Fig. 6a), but the ALS activity of flower buds decreased more than in mature leaves after exposure because ALS enzyme was more active in such rapidly growing tissues such as flower buds. This result confirms that rapeseed is very sensitive to a sub-lethal rate of TBM, and ALS activity is inhibited by the absorbed and translocated TBM. Application of TBM also reduced the content of leaf chlorophyll and the photosynthesis rate (Fig. 6b, c). Moreover, the content of key intermediates of energy metabolism, including pyruvate (the substrate of ALS) and soluble sugar (Fig. 6d, e), also decreased within several days after treatment. In comparison to the common TBM-S genotype SP2F, the TBM-resistant mutant DS3 (TBM-R) showed higher ALS activity, and the photosynthesis rate and contents of chlorophyll, pyruvate, and soluble sugar were less affected (Fig. 6) under the TBM treatment of 1.0 μg per plant, which cannot elicit MS in TBM-R (Fig. 1). Thus, we assumed that these traits accompanying ALS inhibition, including destruction of plastids, depression of photosynthesis, and deficiency of carbohydrates, are also important physiological traits associated with the male sterility induced by the gametocide TBM. We also found that the ethylene release rate by the TBM-treated flower buds increased significantly on 3 and 5 DAT (Fig. 6f), and this suggests the possible occurrence of cell death by the ethylene signal pathway.

### Analyses of differential gene expression in response to ALS-inhibiting herbicides

**Fig. 7:**
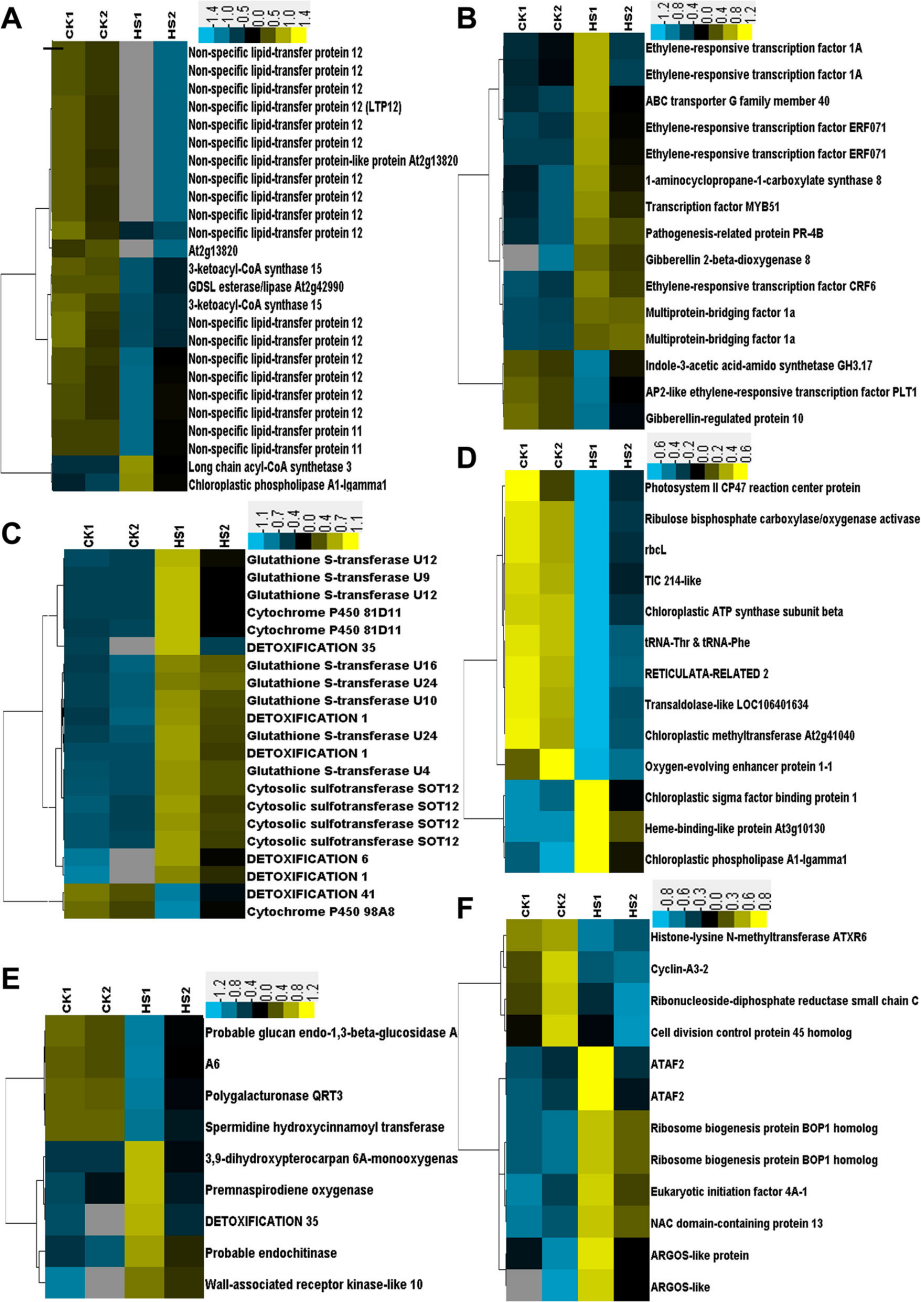
Heatmaps of log-transformed expression level of five groups of selected DETs in the samples. Samples HS1 and HS2 represent the replicates of the TBM treatment and samples CK1 and CK2 represent the replicates of the control. **a** Group of lipid metabolism. **b** Group of hormone response. **c** Group of metabolic detoxification. **d** Group of chloroplast components. **e** Group of cell wall formation. **f** Group of cell division and growth. The detailed gene information is listed in Additional file 2: Dataset S1

The clean RNA-seq reads were mapped by the Bowtie software based on *Brassica* sequences in Gene Index Databases (URL: http://compbio.dfci.harvard.edu/tgi) and NCBI databases after DGE tags profiling of two groups of cDNA libraries of young flower buds on an Illumina Solexa sequencing platform. We identified approximately ninety thousand genes for each sample. We normalized the tag distribution by TPM in each library, and then 363 DETs (200 upregulated and 163 downregulated; Additional file 2: dataset S1) were extracted. The top 50 up- and downregulated genes are listed in Tables 1 and 2. Some genes that were downregulated encode functionally important proteins, such as glucan endo-1,3-beta-glucosidase A6, polygalacturonase QRT3, non-specific lipid-transfer protein LTP11 and LTP12, spermidine hydroxycinnamoyl transferase (SHT), fatty acyl-CoA reductase 2 (MS2), histone-lysine N-methyltransferase ATXR6, indole-3-acetic acid-amido synthetase GH3.17, and gibberellin-regulated protein 10 (GASA10). Meanwhile, some other genes encoding autophagy-related protein ATG8a [20], 1-aminocyclopropane-1-carboxylate synthase 8 (ACS8), ethylene-responsive factor 1A (ERF1A) and RAP2-3, Detoxification 1 (DTX1), DTX6, and DTX35 (or multidrug and toxin efflux family transporter FLOWER FLAVONOID TRANSPORTER [21]), and the glutathione S-transferase family (U9, U10, U11, U12, U16, and U24) were upregulated. The expression of some DETs in five groups related to lipid metabolism, metabolic detoxification, photosynthesis and plastid constitution, hormone responses, cell division and growth, and cell wall construction, are shown in heatmaps (Fig. 7) by the relative level transformed from their TPM values in the four samples.

**Table 1:** The top 50 genes being upregulated by TBM treatment

Accession	Fold change	Gene
FG559204	343.47	Protein DETOXIFICATION 1 (DTX1)
TC164460	245.31	Serine/threonine-protein kinase RAD53
TC161749	177.03	Cytosolic sulfotransferase 12 (SOT12)
TC161707	109.57	Cytosolic sulfotransferase 12 (SOT12)
TC173619	107.97	Protein DETOXIFICATION 1 (DTX1)
TC161747	105.69	Cytosolic sulfotransferase 12 (SOT12)
TC182645	98.67	ABC transporter C family member 3
FG567202	97.45	Cytosolic sulfotransferase 12 (SOT12)
TC168716	95.81	AAA-ATPase At2g18193
TC175294	85.64	Ethylene-responsive transcription factor ERF071
CD828147	77.54	Proline--tRNA ligase, cytoplasmic
ES950391	73.19	ABC transporter C family member 3
EE563787	66.73	Gibberellin 2-beta-dioxygenase 8
TC162388	65.15	Mitochondrial amidoxime-reducing component 1
FG562399	61.24	Ethylene-responsive transcription factor ERF071
TC183183	56.86	Protein DETOXIFICATION 6
FG558108	56.32	NAC domain-containing protein 13
EE448196	52.99	ARGOS-like protein
ES912635	52.67	UDP-glycosyltransferase 73C3
EL624524	50.21	Calcium-transporting ATPase 2, endoplasmic reticulum-type
TC169680	48.78	OPA3-like protein
EV166804	48.59	Protein SRG1
TC171321	47.96	Putative F-box protein At5g55150
TC163384	47.93	ABC transporter B family member 4
TC174670	45.16	External alternative NAD(P)H-ubiquinone oxidoreductase B4, mitochondrial
TC187508	42.47	Ethylene-responsive transcription factor CRF6
TC165449	41.90	Ubiquinol oxidase 3, mitochondrial
TC166236	41.39	UDP-glycosyltransferase 73C5
EE418574	41.02	Transcription factor bHLH130
EE418044	40.59	ABC transporter C family member 3
TC180553	39.97	AAA-ATPase At1g43910
FG565272	39.51	Putative F-box protein At5g55150
TC173139	37.31	Ubiquinol oxidase 1a, mitochondrial
EL625815	36.34	UDP-glycosyltransferase 74E2
TC161751	32.55	Cytosolic sulfotransferase 12
FG566389	31.71	UDP-glycosyltransferase 73C4
FG566278	31.50	Probable inactive poly [ADP-ribose] polymerase SRO3
TC163015	30.51	Indole glucosinolate O-methyltransferase 1
TC188325	29.03	UDP-glycosyltransferase 73C5
FG561297	27.49	Probable inactive poly [ADP-ribose] polymerase SRO3
TC166040	27.35	23.5 kDa heat shock protein, mitochondrial
FG555030	27.09	Putative F-box protein At5g55150
FG565854	26.31	23.5 kDa heat shock protein, mitochondrial
TC170537	26.20	23.5 kDa heat shock protein, mitochondrial
TC169261	25.75	23.5 kDa heat shock protein, mitochondrial
EL625065	24.36	NAC domain-containing protein 13
FG565138	24.18	Putative F-box protein At5g55150
GT074119	24.09	Glutathione S-transferase U12
TC162725	23.85	3-hydroxybenzoate 6-hydroxylase 1
ES988886	23.74	NAC domain-containing protein 13

**Table 2:** The top 50 genes being downregulated by TBM treatment

Accession	Fold change	Gene
EV148555	0.02	Beta-glucosidase 20
TC171681	0.02	Spermidine coumaroyl-CoA acyltransferase
TC164539	0.03	Aquaporin NIP3-1
TC167983	0.05	Beta-glucosidase 20
TC164121	0.05	3-ketoacyl-CoA synthase 15 (KCS15)
TC168401	0.05	Dehydrodolichyl diphosphate synthase 8
TC165695	0.05	Glycine-rich domain-containing protein 2
TC168189	0.05	Glycine-rich domain-containing protein 2
TC176577	0.06	Non-specific lipid-transfer protein 12 (LTP12)
TC168556	0.06	NAC transcription factor 25
TC179510	0.06	Protein *Arabidopsis thaliana* ANTHER 7 (ATA7, Non-specific lipid transfer protein Y.4, At4g28395)
TC165412	0.07	NADP-dependent alkenal double bond reductase P2
TC182788	0.07	Pathogenesis-related protein 5
TC175212	0.07	Spermidine hydroxycinnamoyl transferase
TC169918	0.07	Pathogenesis-related protein 5
TC169758	0.07	Protein HOTHEAD
CD844791	0.07	Putative cysteine-rich repeat secretory protein 17
TC185287	0.07	Polygalacturonase QRT3
TC164597	0.07	Protein COLD-REGULATED 15B, chloroplastic
TC171850	0.08	Probable glucan endo-1,3-beta-glucosidase A6
TC170042	0.08	Probable glucan endo-1,3-beta-glucosidase A6
TC171825	0.08	Non-specific lipid-transfer protein 12
TC173579	0.09	Probable aquaporin NIP7-1
CX190056	0.09	Putative caffeoyl-CoA O-methyltransferase At1g67980
TC169354	0.10	Chalcone synthase 3
TC181187	0.11	Probable glucan endo-1,3-beta-glucosidase A6
TC188816	0.12	Uncharacterized methyltransferase At2g41040, chloroplastic
CX187614	0.12	Probable glucan endo-1,3-beta-glucosidase A6
TC161683	0.12	Chalcone synthase 3
TC163850	0.13	Chalcone synthase 3
TC182159	0.13	Non-specific lipid-transfer protein 12 (LTP12)
TC167903	0.13	Spermidine hydroxycinnamoyl transferase (SHT)
TC185638	0.13	Protein ASPARTIC PROTEASE IN GUARD CELL 1
EV085358	0.14	Dirigent protein 13
EV067241	0.14	Nicotinate phosphoribosyltransferase 1
EV008854	0.14	Probable 6-phosphogluconolactonase 3
CX193525	0.15	Non-specific lipid-transfer protein 12 (LTP12)
TC183728	0.15	Non-specific lipid-transfer protein 12 (LTP12)
TC169260	0.15	Non-specific lipid-transfer protein 12 (LTP12)
EV080638	0.15	Chitinase-like protein 1
TC163412	0.15	Protein DMR6-LIKE OXYGENASE 2
EV087087	0.16	Dirigent protein 12
CX195232	0.16	Uncharacterized protein PF11_0207
TC165145	0.16	Putative caffeoyl-CoA O-methyltransferase At1g67980
TC177265	0.17	NADPH-dependent 1-acyldihydroxyacetone phosphate reductase
CX195872	0.17	Non-specific lipid-transfer protein 12 (LTP12)
TC184478	0.17	Putative dual specificity protein phosphatase DSP8
TC168738	0.17	Cytochrome P450 98A8
TC189006	0.17	Gibberellin-regulated protein 10
EV073392	0.17	L-ascorbate peroxidase 1, cytosolic

**Fig. 8:**
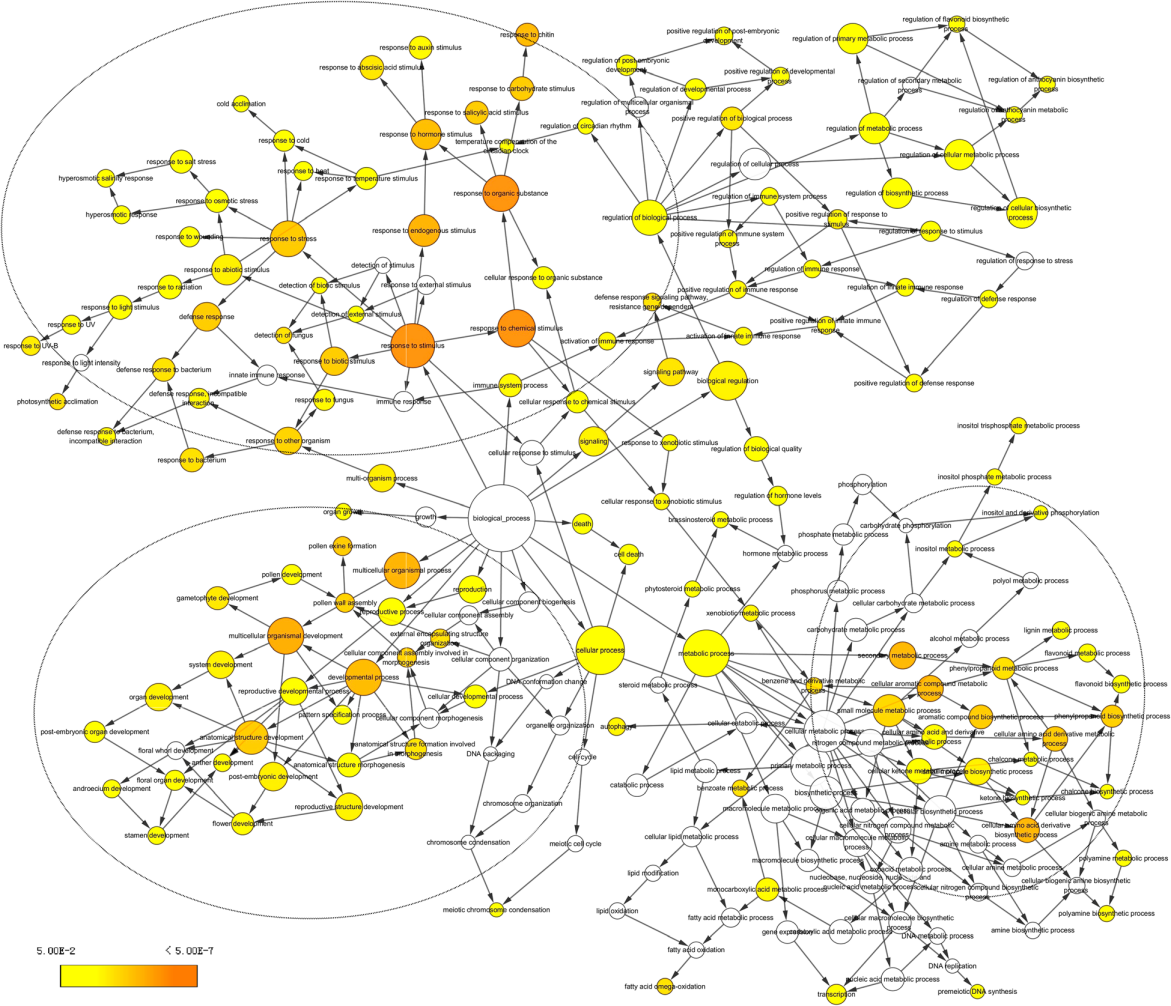
Genetic network of biological processes that were enriched based on the differentially expressed transcripts in the TBM-treated flower transcriptome. Yellow color in a node indicated the significant level

We attempted to match all these DETs to GO terms, and the GOs were enriched by the BiNGO program (Fig. 8). The top 50 up- and downregulated GOs are listed in Tables 3 and 4. It was shown by the genetic network that the main groups of GO terms, including the response to chemical and hormone stimulus (response to stress), developmental processes (pollen wall and pollen exine formation), lipid metabolism, and the cellular amino acid derivative biosynthesis process were significantly influenced by the TBM treatment. As in the amidosulfuron treatment [10], the expression level of the genes in the pathway of BCAAs synthesis via the ALS enzyme showed no obvious change. Interestingly, the genes matching GO:0009693 (ethylene biosynthetic process), including 1-aminocyclopropane1-carboxylate synthase 8 and GO:0009723 (response to ethylene stimulus) multiprotein-bridging factor 1a, ethylene-responsive factor *CRF6* and *ERF071*, and ethylene-responsive transcription factor *RAP2-3*, were upregulated. Meanwhile, the gene 4-substituted benzoates-glutamate ligase *GH3.12* in GO:0009733 (response to auxin stimulus), indole-3-acetic acid-amido synthetase *GH3.17* of GO:0009734 (auxin-activated signalling pathway), and AP2-like ethylene-responsive transcription factor *PLT1* in GO:0009873 (ethylene-activated signalling pathway) were downregulated. The effect on the ethylene biosynthetic process and mediated signalling pathway was supported by an elevated ethylene release rate in Fig. 6f.

**Table 3:** The top 50 GO terms being upregulated by TBM

GO ID	GO_term	S gene number	B gene number	P value of Fisher’s Exact Test
GO:0016131	Brassinosteroid metabolic process	11	18	1.06E-18
GO:0008194	UDP-glycosyltransferase activity	25	142	2.22E-16
GO:0010200	Response to chitin	27	326	4.44E-16
GO:0035251	UDP-glucosyltransferase activity	13	49	5.55E-16
GO:0080118	Brassinosteroid sulfotransferase activity	7	7	5.34E-15
GO:0050502	Cis-zeatin O-beta-D-glucosyltransferase activity	7	11	1.71E-12
GO:0050403	Trans-zeatin O-beta-D-glucosyltransferase activity	7	11	1.71E-12
GO:0009407	Toxin catabolic process	12	89	3.99E-11
GO:0010290	Chlorophyll catabolite transmembrane transporter activity	5	5	6.46E-11
GO:0015431	Glutathione S-conjugate-exporting ATPase activity	5	5	6.46E-11
GO:0052638	Indole-3-butyrate beta-glucosyltransferase activity	5	5	6.46E-11
GO:0071475	Cellular hyperosmotic salinity response	5	5	6.46E-11
GO:0004364	Glutathione transferase activity	12	96	9.91E-11
GO:0016758	Transferase activity, transferring hexosyl groups	13	127	2.10E-10
GO:0009916	Alternative oxidase activity	6	11	2.61E-10
GO:0080043	Quercetin 3-O-glucosyltransferase activity	8	33	5.49E-10
GO:0080044	Quercetin 7-O-glucosyltransferase activity	7	22	8.08E-10
GO:0008146	Sulfotransferase activity	7	23	1.15E-09
GO:0004497	Monooxygenase activity	16	256	2.73E-09
GO:0070370	Cellular heat acclimation	6	16	4.35E-09
GO:0046527	Glucosyltransferase activity	4	4	7.08E-09
GO:0070301	Cellular response to hydrogen peroxide	5	10	1.57E-08
GO:0009408	Response to heat	19	424	2.12E-08
GO:0034605	Cellular response to heat	6	21	2.84E-08
GO:0016757	Transferase activity, transferring glycosyl groups	20	488	3.93E-08
GO:0010016	Shoot morphogenesis	5	12	4.85E-08
GO:0080024	Indolebutyric acid metabolic process	5	13	7.82E-08
GO:0031930	Mitochondria-nucleus signaling pathway	4	6	1.05E-07
GO:0042626	ATPase activity, coupled to transmembrane movement of substances	11	148	1.47E-07
GO:0009061	Anaerobic respiration	4	7	2.42E-07
GO:0050832	Defense response to fungus	15	332	5.86E-07
GO:0010294	Abscisic acid glucosyltransferase activity	5	19	6.75E-07
GO:0051238	Sequestering of metal ion	3	3	7.75E-07
GO:0010508	Positive regulation of autophagy	4	10	1.42E-06
GO:0080046	Quercetin 4′-O-glucosyltransferase activity	5	22	1.50E-06
GO:0080167	Response to karrikin	13	268	1.51E-06
GO:0006979	Response to oxidative stress	16	441	4.29E-06
GO:0043424	Protein histidine kinase binding	6	51	7.46E-06
GO:0003700	Sequence-specific DNA binding transcription factor activity	47	2586	8.09E-06
GO:0009751	Response to salicylic acid stimulus	13	330	1.43E-05
GO:0009414	Response to water deprivation	16	487	1.47E-05
GO:0045333	Cellular respiration	4	17	1.53E-05
GO:0003950	NAD+ ADP-ribosyltransferase activity	4	18	1.96E-05
GO:0007275	Multicellular organismal development	10	210	2.85E-05
GO:0010120	Camalexin biosynthetic process	4	20	3.05E-05
GO:0070825	Micropyle	3	8	4.19E-05
GO:0009651	Response to salt stress	23	1008	7.63E-05
GO:0010224	Response to UV-B	7	113	8.91E-05
GO:0051707	Response to other organism	6	79	9.27E-05
GO:0042631	Cellular response to water deprivation	5	53	0.00012887

Note: There are 437 significant genes in the total 47,474 upregulated genes. S and B gene meant the number of significant genes and the tested gene in a certain pathway, respectively

**Table 4:** The top 50 GO terms being downregulated by TBM

GO Id	GO_term	S gene number	B gene number	P value of Fisher’s Exact Test
GO:0008289	Lipid binding	32	384	0
GO:0006869	Lipid transport	27	388	0
GO:0009505	Plant-type cell wall	28	655	3.87E-14
GO:0009715	Chalcone biosynthetic process	7	16	8.68E-12
GO:0009629	Response to gravity	7	16	8.68E-12
GO:0016210	Naringenin-chalcone synthase activity	7	16	8.68E-12
GO:0016298	Lipase activity	10	63	2.37E-11
GO:0031540	Regulation of anthocyanin biosynthetic process	7	24	2.50E-10
GO:0043169	Cation binding	13	192	1.34E-09
GO:0016788	Hydrolase activity, acting on ester bonds	12	181	7.36E-09
GO:0009813	Flavonoid biosynthetic process	8	58	7.79E-09
GO:0016747	Transferase activity, transferring acyl groups other than amino-acyl groups	11	166	3.15E-08
GO:0016746	Transferase activity, transferring acyl groups	9	107	7.41E-08
GO:0006629	Lipid metabolic process	13	278	1.09E-07
GO:0019953	Sexual reproduction	6	39	3.12E-07
GO:0080088	Spermidine hydroxycinnamate conjugate biosynthetic process	3	3	3.46E-07
GO:0080074	Spermidine:caffeoyl COA N-acyltransferase activity	3	3	3.46E-07
GO:0016410	N-acyltransferase activity	3	3	3.46E-07
GO:0080075	Spermidine:feruloyl COA N-acyltransferase activity	3	3	3.46E-07
GO:0080073	Spermidine:coumaroyl COA N-acyltransferase activity	3	3	3.46E-07
GO:0019915	Lipid storage	6	48	1.11E-06
GO:0080072	Spermidine:sinapoyl COA N-acyltransferase activity	3	4	1.38E-06
GO:0050734	Hydroxycinnamoyl transferase activity	3	4	1.38E-06
GO:0009859	Pollen hydration	4	13	1.64E-06
GO:0004869	Cysteine-type endopeptidase inhibitor activity	5	33	3.39E-06
GO:0009926	Auxin polar transport	7	90	3.63E-06
GO:0010584	Pollen exine formation	6	60	4.22E-06
GO:0004553	Hydrolase activity, hydrolyzing O-glycosyl compounds	13	392	5.09E-06
GO:0010224	Response to UV-B	7	101	7.83E-06
GO:0005788	Endoplasmic reticulum lumen	5	41	1.02E-05
GO:0008610	Lipid biosynthetic process	5	42	1.15E-05
GO:0009705	Plant-type vacuole membrane	8	149	1.16E-05
GO:0009733	Response to auxin stimulus	12	376	1.72E-05
GO:0042409	Caffeoyl-COA O-methyltransferase activity	3	10	4.00E-05
GO:0005576	Extracellular region	7	142	7.08E-05
GO:0003824	Catalytic activity	26	1598	7.46E-05
GO:0031012	Extracellular matrix	4	34	9.47E-05
GO:0005199	Structural constituent of cell wall	3	15	0.000148
GO:0005975	Carbohydrate metabolic process	14	625	0.000162
GO:0016706	Oxidoreductase activity, acting on paired donors	4	42	0.000219
GO:0005506	Iron ion binding	7	175	0.000259
GO:0016832	Aldehyde-lyase activity	3	19	0.000308
GO:0016614	Oxidoreductase activity, acting on CH-OH group of donors	3	22	0.000483
GO:0016765	Transferase activity, transferring alkyl or aryl (other than methyl) groups	2	6	0.000728
GO:0006979	Response to oxidative stress	11	504	0.000992
GO:0009753	Response to jasmonic acid stimulus	8	295	0.001253
GO:0009821	Alkaloid biosynthetic process	3	35	0.001917
GO:0016844	Strictosidine synthase activity	3	38	0.002433
GO:0016491	Oxidoreductase activity	12	663	0.002847

Note: There are 290 significant genes in the 41,162 downregulated genes

**Fig. 9:**
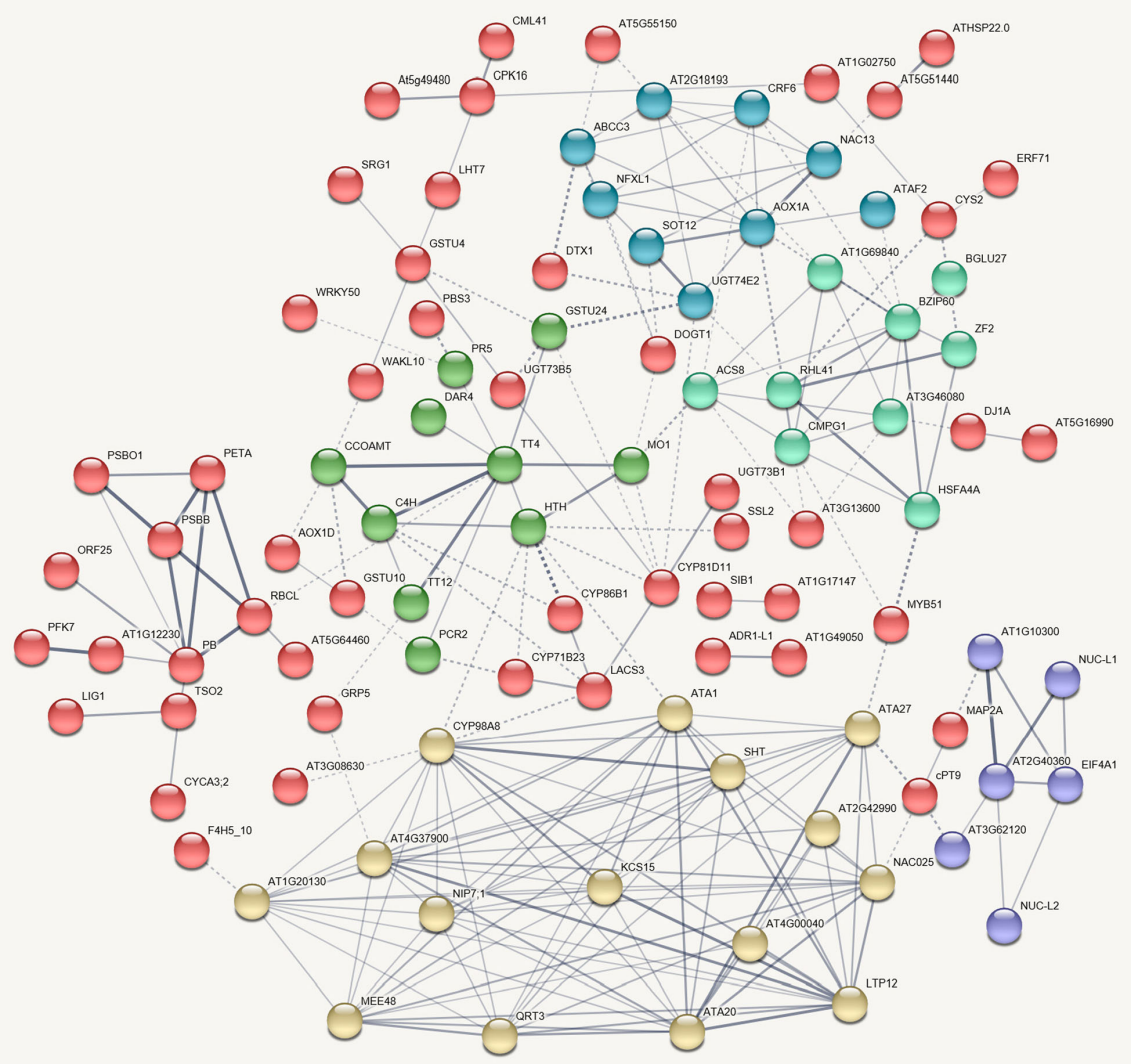
All known and predicted protein-protein association analyses based on STRING Arabidopsis genes homologous to the differentially expressed transcripts. Six groups of proteins are presented by their nodes in different colours. Thickness of edge showed different confidence levels: low (0.15), medium (0.40), high (0.70), or highest (0.90). The details of these proteins can be found in Additional file 3: Table S2

We further showed the possible protein-protein interactions among the DETs by a network produced by STRING. The derived interaction network (Fig. 9) involved 147 proteins, and it could be further divided into several groups. The first group is comprised of 18 proteins, which are all involved in lipid and pollen exine formation. The important members among them include glucanase A6 (MEE48), polygalacturonase QRT3, anther-specific protein ATA7, ATA20 and ATA27, short-chain dehydrogenase reductase tapetum 1 (ATA1), 3-Ketoacyl-CoA synthase 15 (KCS15), Type III polyketide synthase C (At4g00040), non-specific lipid-transfer protein 12 (LTP12), spermidine hydroxycinnamoyl transferase (SHT), aquaporin NIP7-1 (NIP7;1), glycine-rich domain-containing protein 2 (GRDP2/At4g37900, involved in development and stress responses [22]), and cytochrome P450 98A8. The second group is about defence response and signalling, consisting of cytosolic sulfotransferase (SOT) 12, detoxification DTX1, UDP-glucosyl transferase UGT73B1, UGT73B3 and UGT73B5, PHYTOCYSTATIN 2 (CYS2), sigma factor binding protein SIB1, cysteine-rich receptor-like protein kinase CRK5, pathogenesis-related protein PR-4B, 2-Alkenal reductase AT5G16990, glutathione S-transferase (GST) GSTU4, and GSTU9, detoxifying efflux carrier DTX35, and transcription factor MYB51. The third group mainly includes hormone-related pathways, including fibberellin-regulated protein 10, GH3.12, indole-3-acetic acid-amido synthetase GH3.17, 1-Aminocyclopropane-1-carboxylate synthase 8 (ACS8), ethylene-responsive ERF1, ERF071, RAP2-3, CRF6, and multiprotein-bridging factor 1a. Ethylene response factors comprise a large family of transcription factors that regulate numerous biological processes, including growth, development, and response to environmental stresses. It was shown that some other proteins may also play important roles in this regulation network, for example, CCOMAT, TT4, C4H, TT12, CYP86B1, and LACS3 among the pathway of flavonoid metabolism. These abnormal transcriptional regulations are in accordance with the functional abnormalities of pollen wall formation, lipid metabolism, plastid structure, and tissue autophagy mentioned above.

### Expression analysis of *ALS* and other important genes detected by real-time PCR

**Fig. 10:**
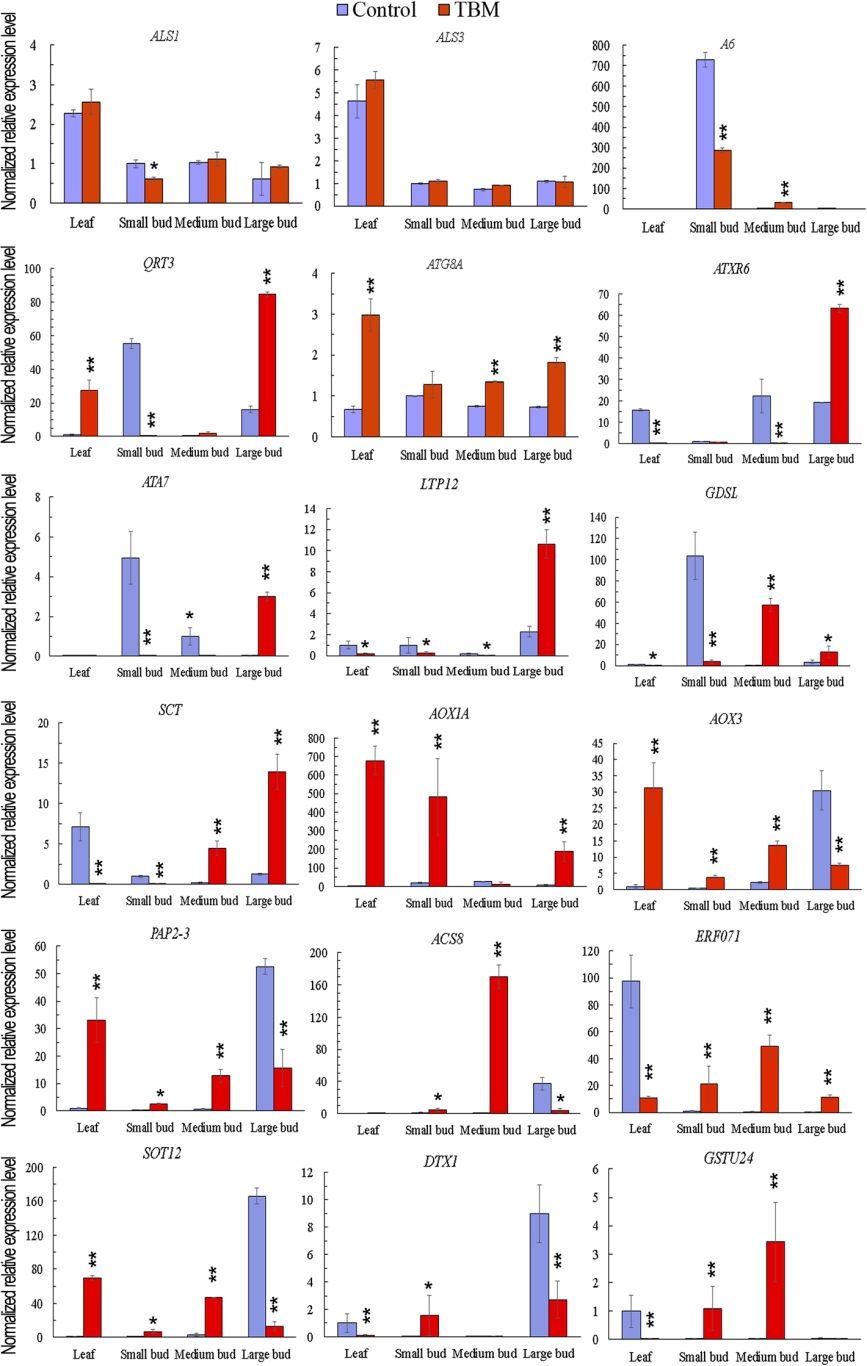
Relative gene expression in the indicated tissues of *ALS* genes and 16 differentially expressed transcripts. The y-axes represent the relative gene expression level, which is the −2^ΔCt^ value of qPCR compared to the internal control gene *BnActin7*. Note: * and ** indicate significant differences at the 95 and 99% confidence levels by Student’s *t*-test. Error bars represent the standard deviation

The expression levels of *ALS1* and *ALS3*, represented by a fold change among the treatment and control in qPCR, were not severely affected in the TBM-treated plants (Fig. 10), except that *ALS1* was only slightly elevated in small buds. This result meant that sub-lethal TBM exposure did not greatly perturb the *ALS* expression, although the enzyme activity was obviously inhibited. The other 16 interesting DETs (Fig. 10**)** were also chosen for qPCR analysis. The results showed that, in the small buds and medium buds, the up/down regulation of the selected DETs were in line with their TPM values in RNA-seq data (Fig. 10). Two genes that encode glucanase A6, and QRT3, which play important roles in microspore release from tetrads, were downregulated. In the small flower buds of the treated plants, non-specific lipid-transfer protein LTP12 and LtpY.4/anther protein ATA7, which were predominantly expressed at the early stage of anther development [23], GDSL-like lipase AT1G20130, spermidine coumaroyl-CoA acyltransferase (SCT), and chloroplastic psbB were also downregulated. Histone-lysine N-methyltransferase ATXR6, which may act as a positive regulator of the G1-S transition in the cell cycle, was inhibited in the TBM treatment. All eight tested genes related to chemical stimulus, stress response, ethylene signal, and autophagy, including *SOT12, DTX1, GSTU24, AOX1A, AOX3, PAP2-3, ACS8, ERF071,* and *ATG8a*, were greatly activated by the TBM treatment. Unfortunately, we did not analyse anther and pistil separately, so we do not know which genes are expressed anther-specifically.

## Discussion

### Inhibition of ALS activity is necessary but not enough for TBM to induce MS

It is well known that CIMS involves multiple metabolic pathways, but which metabolic pathway plays a key role is hard to determine. It seemed that TBM would become the first gametocide whose plant target enzyme and key biological pathways had been revealed because previous studies had produced extensive knowledge about ALS-inhibiting herbicides. ALS is the direct target of various SU and imidazolinone herbicides, including TBM [1, 2]. In the present study, even trace amounts of TBM can obviously depress the ALS activity of rapeseed plants. The ALS activity, and other symptoms such as chlorophyll and photosynthesis, was not affected in the TBM-resistant genotype TBM-R. In another study, the transgenic expression of SU-resistant mutant *csr1-1D* could reverse the male sterile phenotype induced by TBM in both rapeseed and Arabidopsis [17]. Therefore, the inhibition of ALS activity seemed to be a precondition for TBM and other SU gametocides to induce plant MS.

However, ALS did not seem to be the only site that affected the capability of SU gametocides because we found that triazolopyrimidine and pyrimidinylthiobenzoate herbicides did not cause rapeseed male sterility, though they also inhibited the activity of the ALS enzyme and plant growth [5]. Thus, the inhibition of ALS activity is not a guaranty of plant MS. Resistance to ALS inhibitors appears complex and involves a set of unknown non-target-site resistance loci from different chemical families [24]. Except for ALS enzymes, metabolic detoxification mediated by cytochrome P450-monooxygenase [25] and GST conjugation [26], metabolic replenishment via a regulatory protein kinase general control non-derepressible-2 (GCN2) [18, 27], and amino acid recycle via protein turn-over [10] may also contribute to the biological tolerance of SU herbicides. GCN2 conferring amino acid balance and BCAA recycle caused by autophagy were activated by TBM-induced BCAA starvation and independently contributed to TBM tolerance in plants [18]. Arabidopsis *gcn2* mutant also showed a lower inhibition of photosynthesis by glyphosate [28]*.* Duhoux et al., [29] found that four genes, including two cytochromes P450, one glycosyl-transferase and one *GST*, were non-target-site resistance markers, whose combined expression levels could reliably identify resistant plants in ryegrass. In the present study, the TBM treatment also resulted in autophagy in the tapetal cells and microspores, letting them degrade quickly at early development stages, as amidosulfuron did [10]. Detoxification was also suggested by the elevated expression of dozens of genes encoding the proteins of Detoxification 1, Cytochrome P450, and the GST family. Therefore, the results of our present study suggest that except for the ALS enzyme and BCAA biosynthesis, some new metabolic pathways need to be studied to address the cause of phytotoxicity, including the MS phenotype.

### Metabolic detoxification response to TBM exposure

It was found that IM-treated *Arabidopsis* expressed detoxification genes at early time points that encode steroid SOT, 1-aminocyclopropane-1-carboxylic acid oxidase, glycosyltransferase, GST, cytochrome P450, ATP-binding cassette (ABC) transporter, multidrug and toxin extrusion (MATE) and alternative oxidase (AOX) protein families [14]. Some new genes implicated in detoxification, including *SOT12*, *At2g41730* and several components of alternative respiratory pathway, MATE transporter *DTX4*, *DTX3* and *DTX1*, were revealed [14].

Similarly, we found that the expression of some detoxification and defence-related genes, for example, drug metabolism by the cytochrome P450 family, SOT12, and alternative oxidase AOX1A and AOX3, were also induced by TBM. Mitochondrial ubiquinol oxidase AOX1A and AOX3 are expressed in the mitochondria and are known to respond to various stresses [30]. Fermentative metabolism was also induced in pea plants by ALS-inhibiting herbicide, although oxidative stress seemed not related to the mode of action of herbicides [31]. SOT12 is a brassinosteroid SOT and is produced in response to NaCl stress, ABA, and salicylic acid [32]. The abovementioned data about the glycosyltransferase, GST, cytochrome P450, ABC transporter, MATE, AOX, and SOT indicate that one major response towards TBM treatment is the induction of detoxification-related genes.

### Plastid destruction and lipid abnormality is associated with MS induced by ALS inhibitors

SU herbicides may damage the photosynthetic apparatus [33] because plant ALS proteins are located in plastids and chloroplasts [34]. We found that the photosynthetic rate and chlorophyll content were obviously affected in the several days after the sub-lethal dose of the TBM treatment, similar to the effects of another gametocide amidosulfuron [10]. The abnormality of chloroplasts and tapetal elaioplasts caused by SU gametocides had been previously reported by us [10]. Zabalza et al. [35] found that both inhibitors of KARI and ALS induced growth arrest and photosynthesis inhibition. The exposure of *A. thaliana* plants to herbicide IM strongly affected chlorophyll synthesis and increased reactive oxygen species (ROS) [7, 8]. The exposure of rice to IM damaged lipid membranes and affected the transcription of genes involved in photosynthesis and sugar metabolism [7, 36]. Carbohydrate metabolism may be blocked in either treatments of TBM, amidosulfuron [10] or MES exposure [9] because they led to a decrease in the soluble sugar contents in leaves and flower buds. The results of Sun et al., [36] showed that the PSII system was severely damaged, and the expression of many photosynthesis-related genes decreased in the IM-treated plants.

The destruction of chloroplasts and the incapability of photosynthesis seemed to be the main effects of the TBM treatments and other ALS-inhibiting gametocides [7–10, 32, 36]. Developing microspores need a large supply of photoassimilates [37], such as starch and other carbohydrates. The deprivation of the anther sugar content seemed to be one important factor in stress-induced MS (Review in [37]). Therefore, the deprivation of photosynthesis and the destruction of plastids also contribute to the MS induced by ALS-inhibitors.

### Abnormal pollen coat of gametocide-induced MS

The formation and dissolution of the callose wall covering MMC and tetrad microspores are important processes during and after plant meiosis [38]. Both microscopic and ultramicroscopic observations in this study demonstrated an abnormal callose layer surrounding tetrad microspores. The components of the wall are mainly β-1, 3-glucans and pectin. The downregulation of the tapetum marker gene *glucanase A6* and *QRT3* prove that the callose layer covering MMC and tetrad microspores is affected by TBM treatment. The downregulation of the other genes related to cell wall construction (Fig. 6) also implied abnormalities in pollen wall construction.

Numerous studies using mutants have revealed that lipids play important roles not only in the formation of thylakoid membranes but also in the folding and assembly of the protein subunits in photosynthetic complexes. In addition, studies on thylakoid membranes have also provided critical information on the association of lipids with photosynthetic complexes and their activities [39]. Both the TBM and amidosulfuron treatment [10] resulted in defective elaioplasts and pollen exine. Defective plastids will impair the tapetal secretion of such materials as flavonoid and lipid compounds and finally affect the formation of pollen coat [40]. AT1G20130 is a GDSL-like lipase that is highly expressed during microspore mitosis, with a dramatic decline during pollen maturation (according to UniProt annotation). LTP11 and LTP12 are non-specific lipid-transfer proteins that transfer phospholipids as well as galactolipids across membranes. They may play a role in wax or cutin deposition in the cell walls of expanding epidermal cells and certain secretory tissues (according to UniProt annotation).

In addition, some plant secondary metabolites, such as phenylpropanoid and flavonoids, also affect the pollen coat construction. SHT catalyses the biosynthesis of trihydroxycinnamoyl spermidine derivatives that accumulate on the pollen coat [41]. Plant type III polyketide synthase catalyses the condensation of malonyl-CoA units with various CoA ester starter molecules to generate a diverse array of natural products, including long-chain alkyl alpha-pyrones. The protein belongs to the chalcone synthase superfamily and generates backbones of a variety of plant secondary metabolites, including chalcones, stilbenes, biphenyls, etc. [42]. The upregulation of such cytochrome P450 members as *CYP71B23*, *CYP 81D11* and *CYP72A219*, and the downregulation of *CYP 98A8* and *CYP 86B1*, suggested an influence on the pathway of phenylpropanoid and flavonoid metabolism.

### Mode of action for gametocide TBM

**Fig. 11:**
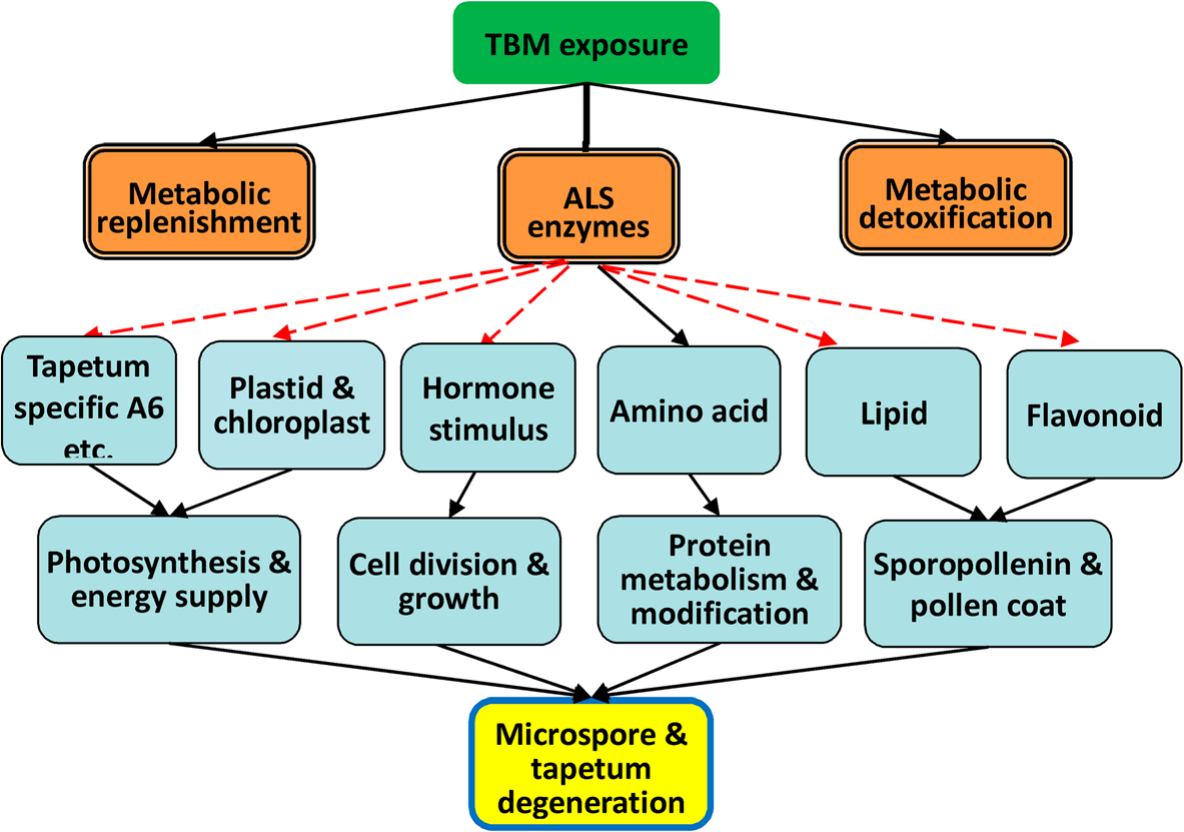
Scheme of mode of action for SU gametocides (modified from Liu et al., 2017). Exposure to TBM causes inhibition of ALS enzymes, metabolic detoxification, and metabolic replenishment. Inhibition of ALS activity disturbs amino acid metabolism and then destroys plastids and chloroplasts. Then, the expression of some tapetum preferential genes and the synthesis of lipids, flavonoids, and pollen coat materials is affected. Consequently, photosynthesis, energy and nutrition supply, cell division and development, and protein metabolism are all influenced by the treatment

As another gametocide amidosulfuron did [10], TBM also showed multi-effects on rapeseed growth and development. Although TBM and amidosulfuron treatment elicited somewhat different transcriptional regulation in rapeseed, we could find similar DET categories between the two transcriptomes, such as lipid metabolism, chloroplastic components, cell cycle, and cell wall construction. The available data in our studies and previous studies [7–10, 32, 36] suggested that the depression of photosynthesis, the block of the cell cycle, the depletion of pollen coat materials, and the elevation in ethylene are the major phytotoxic effects of TBM. These abnormalities may directly/indirectly induce autophagy and cell death in tapetal cells and microspores. Thus, the mode of action for TBM is similar to that of amidosulfuron [10], as shown in Fig. 11. Exposure to TBM causes the inhibition of ALS enzymes, metabolic detoxification, and metabolic replenishment. The inhibition of ALS activity disturbs amino acid metabolism and then destroys plastids and chloroplasts. Then, the expression of some tapetum preferential genes and synthesis of lipid, flavonoid, and pollen coat materials are affected.

## Conclusion

The results indicate that TBM treatment leads to the abnormal transcriptional regulation of some important pathways, especially those related to plastids, photosynthesis, lipid metabolism, and ethylene stimulus. These abnormalities, proved by the changes of several important physiological traits, will disturb the development of tapetal cells and microspores, and consequently, the MS phenotype occurred. The finding of those important pathways associated with the MS induced by TBM apart from ALS is also a useful reference to study the phytotoxic effect of other ALS-inhibitors.

## Additional files

Additional file 1: **Table S1.** Primers for *ALS* genes and 16 selected differentially expressed transcripts. (DOC 38 kb)

Additional file 2: **Dataset S1.** The list of DETs between TBM-treatment and the control. The DETs were selected with FDR < 0.05 and fold change ≥2. The gene name and symbol, TPM value, fold change, *p* value, gene sequence, GO term, KEGG pathway, BLAST hit in the NCBI database, and functional annotation in the UniProt database were included. (XLSX 163 kb)

Additional file 3: **Table S2.** The symbols and names of all proteins/genes used in STRING database. (DOC 135 kb)

## Abbreviations

ALS: Acetolactate synthase
BCAA: Branched-chain amino acids
CIMS: Chemically induced male sterility
DAT: Days after treatment
DET: Differential expression transcript
DGE: Digital gene expression
MES: Monosulfuron ester sodium
MMC: Microspore mother cell
TEM: Transmission electron microscopy

## References

[1] McCourt A, Pang SS, King-Scott J, Guddat LW, Duggleby RG (2006). Herbicide-binding sites revealed in the structure of plant acetohydroxyacid synthase. Proc Natl Acad Sci U S A.

[2] Zhou Q, Liu W, Zhang Y, Liu KK (2007). Action mechanisms of acetolactate synthase-inhibiting herbicides. Pest Biochemi Physiol.

[3] Yu C, Hu S, He P, Sun G, Zhang C, Yu Y (2006). Inducing male sterility in *Brassica napus* L. by a sulphonylurea herbicide tribenuron-methyl. Plant Breed.

[4] Yu CY, Dong JG, Hu SW, He PR (2009). Efficiency of a novel gametocide amidosulfuron on rapeseed (*Brassica napus*). Plant Breed.

[5] Yu C, He B (2014). Evaluation of male-sterility induction effect of various amino acid biosynthesis inhibiting-herbicides on rapeseed (*Brassica napus*). Acta Agronom Sin.

[6] Yu CY, Dong JG, Hu SW, Xu AX (2017). Exposure to trace amounts of sulfonylurea herbicide tribenuron-methyl causes male sterility in 17 species or subspecies of cruciferous plants. BMC Plant Biol.

[7] Qian H, Wang R, Hu H, Lu T, Chen X, Ye H, Liu W, Fu Z (2011). Enantioselective phytotoxicity of the herbicide imazethapyr and its effect on rice physiology and gene transcription. Environ Sci Technol.

[8] Qian H, Li Y, Sun C, Lavoie M, Xie J, Bai X, Fu Z (2015). Trace concentrations of imazethapyr (IM) affect floral organs development and reproduction in *Arabidopsis thaliana*: IM-induced inhibition of key genes regulating anther and pollen biosynthesis. Ecotoxicology..

[9] Li Z, Cheng Y, Cui J, Zhang P, Zhao H, Hu S (2015). Comparative transcriptome analysis reveals carbohydrate and lipid metabolism blocks in *Brassica napus* L male sterility induced by the chemical hybridization agent monosulfuron ester sodium. BMC Genomics.

[10] Liu XQ, Yu CY, Dong JG, Hu SW, Xu AX (2017). Acetolactate synthase-inhibiting gametocide amidosulfuron causes chloroplast destruction, tissue autophagy, and elevation of ethylene release in rapeseed. Front Plant Sci.

[11] Chakrabarty SK, Maity A, Yadav JB (2015). Influence of cyto-sterility sources of female line on seed quality of Indian mustard (*Brassica juncea* L. Czern & Coss.) in relation to storage period. Plant Breed.

[12] Dey SS, Bhatia R, Parkash C, Sharma S, Dabral M, Mishra V, Bhardwaj I, Sharma K, Kumar SV, Kumar R (2017). Alteration in important quality traits and antioxidant activities in *Brassica oleracea* with Ogura cybrid cytoplasm. Plant Breed.

[13] Xia S, Wang Z, Zhang H, Hu K, Zhang Z, Qin M, Dun X, Yi B, Wen J, Ma C, Shen J, Fu T, Tu J (2016). Altered transcription and neofunctionalization of duplicated genes rescue the harmful effects of a chimeric gene in *Brassica napus*. Plant Cell.

[14] Manabe Y, Tinker N, Colville A, Miki B (2007). CSR1, the sole target of imidazolinone herbicide in *Arabidopsis thaliana*. Plant Cell Physiol.

[15] Glombitza S, Dubuis PH, Thulke O, Welzl G, Bovet L, Götz M, Affenzeller M, Geist B, Hehn A, Asnaghi C, Ernst D, Seidlitz HK, Gundlach H, Mayer KF, Martinoia E, Werck-Reichhart D, Mauch F, Schäffner AR (2004). Crosstalk and differential response to abiotic and biotic stressors reflected at the transcriptional level of effector genes from secondary metabolism. Plant Mol Biol.

[16] Das M, Reichman JR, Haberer G, Welzl G, Aceituno FF, Mader MT, Watrud LS, Pfleeger TG, Gutierrez RA, Schaffner AR, Olszyk DM (2010). A composite transcriptional signature differentiates responses towards closely related herbicides in *Arabidopsis thaliana* and *Brassica napus*. Plant Mol Biol.

[17] Zhao L, Jing X, Chen L, Liu Y, Su Y, Liu T, Gao C, Yi B, Wen J, Ma C, Tu J, Zou J, Fu T, Shen J (2015). Tribenuron-methyl induces male sterility through anther-specific inhibition of acetolactate synthase leading to autophagic cell death. Mol Plant.

[18] Zhao L, Deng L, Zhang Q, Jing X, Ma M, Yi B, Ma C, Tu J, Fu T, Shen J (2018). Autophagy contributes to sulfonylurea herbicide tolerance via GCN2-independent regulation of amino acid homeostasis. Autophagy..

[19] Hu M, Pu H, Long W, Gao J, Qi C, Zhang J, Chen S (2015). Enzymatic characteristics of acetolactate synthase mutant S638N in Brassica napus and its resistance to ALS inhibitor herbicides. Acta Agronom Sin.

[20] Pu Y, Luo X, Bassham DC (2017). TOR-dependent and–independent pathways regulate autophagy in *Arabidopsis thaliana*. Front Plant Sci.

[21] Thompson EP, Wilkins C, Demidchik V, Davies JM, Glover BJ (2010). An Arabidopsis flavonoid transporter is required for anther dehiscence and pollen development. J Exp Bot.

[22] Ortega-Amaro MA, Rodriguez-Hernandez AA, Rodriguez-Kessler M, Hernandez-Lucero E, Rosales-Mendoza S, Ibanez-Salazar A, Delgado-Sanchez P, Jimenez-Bremont JF (2014). Overexpression of AtGRDP2, a novel glycine-rich domain protein, accelerates plant growth and improves stress tolerance. Front Plant Sci.

[23] Boutrot F, Chantret N, Gautier MF (2008). Genome-wide analysis of the rice and Arabidopsis non-specific lipid transfer protein (nsLtp) gene families and identification of wheat *nsLtp* genes by EST data mining. BMC Genomics.

[24] Scarabel L, Pernin F, Délye C (2015). Occurrence, genetic control and evolution of non-target-site based resistance to herbicides inhibiting acetolactate synthase (ALS) in the dicot weed *Papaver rhoeas*. Plant Sci.

[25] Liu C, Liu S, Wang F, Wang Y, Liu K (2012). Expression of a rice CYP81A6 gene confers tolerance to bentazon and sulfonylurea herbicides in both Arabidopsis and tobacco. Plant Cell Tiss Org.

[26] Andrews CJ, Skipsey M, Townson JK, Morris C, Jepson I, Edwards R (1997). Glutathione transferase activities toward herbicides used selectively in soybean. Pestic Sci.

[27] Zhang Y, Wang Y, Kanyuka K, Parry MAJ, Powers ST, Halford NG (2008). GCN2-dependent phosphorylation of eukaryotic translation initiation factor-2α in Arabidopsis. J Exp Bot.

[28] Faus I, Zabalza A, Santiago J, Nebauer SG, Royuela M, Serrano R, Gadea J (2015). Protein kinase GCN2 mediates responses to glyphosate in Arabidopsis. BMC Plant Biol.

[29] Duhoux A, Carrère S, Gouzy J, Bonin L, Délye C (2015). RNA-Seq analysis of rye-grass transcriptomic response to an herbicide inhibiting acetolactate-synthase identifies transcripts linked to non-target-site-based resistance. Plant Mol Biol.

[30] Fiorani F, Umbach AL, Siedow JN (2005). A study of Arabidopsis AOX1a transgenic plants. Plant Physiol.

[31] Zabalza A, Gaston S, Sandalio LM, Rio L-D, Royuela M (2006). Oxidative stress is not related to the mode of action of herbicides that inhibit acetolactate synthase. Environ Experim Bot.

[32] Baek D, Pathange P, Chung JS, Jiang J, Gao L, Oikawa A, Hirai MY, Saito K, Pare PW, Shi H (2010). A stress-inducible sulphotransferase sulphonates salicylic acid and confers pathogen resistance in Arabidopsis. Plant Cell Environ.

[33] Saja D, Rys M, Stawoska I, Skoczowski A (2016). Metabolic response of cornflower (*Centaurea cyanus* L.) exposed to tribenuron-methyl: one of the active substances of sulfonylurea herbicides. Acta Physiol Plant.

[34] Shimizu M, Goto M, Hanai M, Shimizu T, Izawa N, Kanamoto H, Tomizawa KI, Yokota A (2008). Kobayashi H selectable tolerance to herbicides by mutated acetolactate synthase genes integrated into the chloroplast genome of tobacco. Plant Physiol.

[35] Zabalza A, Zulet A, Gil-Monreal M, Igal M, Royuela M (2013). Branched-chain amino acid biosynthesis inhibitors: herbicide efficacy is associated with an induced carbon–nitrogen imbalance. J Plant Physiol.

[36] Sun C, Chen S, Jin Y, Song H, Ruan S, Fu Z, Asad MA, Qian H (2016). Effects of the herbicide imazethapyr on photosynthesis in PGR5- and NDH-deficient *Arabidopsis thaliana* at the biochemical, transcriptomic, and proteomic levels. J Agric Food Chem.

[37] De Storme N, Geelen D (2014). The impact of environmental stress on male reproductive development in plants: biological processes and molecular mechanisms. Plant Cell Environ.

[38] Zhang ZB, Zhu J, Gao JF, Wang C, Li H, Zhang HQ, Zhang S, Wang DM, Wang QX, Huang H, Xia HJ, Yang ZN (2007). Transcription factor AtMYB103 is required for anther development by regulating tapetum development, callose dissolution and exine formation in Arabidopsis. Plant J.

[39] Kobayashi K, Endo K, Wada H (2016). Roles of lipids in photosynthesis. Subcell Biochem.

[40] Dobritsa A, Lei Z, Nishikawa S, Urbanczyk-Wochniak E, Huhman DV, Preuss D, Sumner LW (2010). LAP5 and LAP6 encode anther-specific proteins with similarity to chalcone synthase essential for pollen exine development in Arabidopsis. Plant Physiol.

[41] Elejalde-Palmett C, de Bernonville TD, Glevarec G, Pichon O, Papon N, Courdavault V, St-Pierre B, Giglioli-Guivarch N, Lanoue A, Besseau S (2015). Characterization of a spermidine hydroxycinnamoyltransferase in *Malus domestica* highlights the evolutionary conservation of trihydroxycinnamoyl spermidines in pollen coat of core Eudicotyledons. J Exp Bot.

[42] Abe I, Morita H (2010). Structure and function of the chalcone synthase superfamily of plant type III polyketide synthases. Nat Prod Rep.

