# Supplementary material for: How exposure to ALS-inhibiting gametocide tribenuron-methyl induces male sterility in rapeseed
Source: BMC Plant Biol. 2019 Apr 2;19:124. doi: 10.1186/s12870-019-1722-1 (PMC6444545; doi:10.1186/s12870-019-1722-1)
Supplement: Supplementary file 1 — Table S1. Primers for ALS genes and 16 selected differentially expressed transcripts. (DOC 38 kb) [file 12870_2019_1722_MOESM1_ESM.doc]

Additional file 1

**Table S1. Primers for *ALS* genes and 16 selected differentially expressed transcripts.**

| Gene name | Forward sequence (5’-3’) | Reverse sequence(5’-3') | |
| --- | --- | --- | --- |
| *ALS1* | ACCCGTCAATGTCGCACCTCCTTC | | CCCGAAGTGGCTATGCAGATTCCC |
| *ALS3* | GCTATTCAGACAATGCTGGATACACC | | CTGGGAACAAACCAAAAGCAGTACA |
| *ATG8a* | TCTTCTTCGGAGTCCAATCG | | CTCTGTCAGGGTACTTCTCTC |
| *A6* | CAATCTTGCTTGCCAATGCTATC | | TGCGAACCAAACACAGAAAGTC |
| *QRT3* | ATATCAGGACAAGCAAAC | | CGGTATAATCCAAGTAAGAA |
| *LTP12* | GTGTTTCAGTTCCCTACC | | CATCTCTAGCCTCCTTCA |
| *ATA7* | TCTGTTGGAATGGACTGTT | | ACTCGTCACTAATACAATAAGC |
| *ATXR6* | GTAGAGGCTGATAGGTGTATT | | ATGCTGTCTCCATCATAGTT |
| *RAP2-3* | GGTTGATTTATGGATGTTTGATG | | GCGGTTTATATTACAGCCTTTA |
| *ACS8* | TACTCTAACGATGATATTGTTG | | AGGTAGTTCTTGGTGAAG |
| *AOX1A* | TACTGAATGGAAGTGGAAT | | AATAAGCCAATCTGTCAAG |
| *AOX3* | CCTCCGACTAAGATTACTAA | | GGTTTGTGATGTTTGGTTA |
| *ERF071* | CAAACTCAATTTCCCTAACG | | ATCGCAAGCATTCATCAA |
| *SOT12* | GTGAGATTGTGAAGTTGTGTAG | | AAGCGTTAGACTCTATTCCATT |
| *DTX1* | TGGTTTAGTGGGTGCTTT | | AGTATGTGTAGGTTCCGATT |
| *GSTU24* | GTCGGAGAAGGTCACTAC | | CTCACAGAATCACATTATTAAGC |
| *GDSL* | GGTAGTGGTTACATTCCT | | TTGTCCTACTAACTTCTTCA |
| *SCT* | GGATTCTACGGTAACGCCTAC | | AGAGATTGATGCTTCTTGGAGTT |
| *BnActin7* | CGCGCCTAGCAGCATGAA | | GTTGGAAAGTGCTGAGAGATGCA |
