# Supplementary material for: How exposure to ALS-inhibiting gametocide tribenuron-methyl induces male sterility in rapeseed
Source: BMC Plant Biol. 2019 Apr 2;19:124. doi: 10.1186/s12870-019-1722-1 (PMC6444545; doi:10.1186/s12870-019-1722-1)
Supplement: Supplementary file 3 — Table S2. The symbols and names of all proteins/genes used in STRING database. (DOC 135 kb) [file 12870_2019_1722_MOESM3_ESM.doc]

Additional file 3

**Table S2 The symbols and names of all proteins/genes used in STRING database.**

| Short name | Full name and function |
| --- | --- |
| AT1G02750 | Drought-responsive family protein (221 aa) |
| UGT74E2 | Uridine diphosphate glycosyltransferase 74E2 |
| F4H5_10 | Phospholipase A1-Igamma1 |
| LIG1 | DNA ligase 1 |
| NFXL1 | NF-X-like 1 |
| AT1G10300 | Nucleolar GTP-binding protein |
| AT1G12230 | Transaldolase (427 aa) |
| PCR2 | PLANT CADMIUM RESISTANCE 2 |
| SRG1 | Protein SRG1 (358 aa) |
| AT1G17147 | VQ motif-containing protein |
| GSTU24 | Glutathione S-transferase TAU 24 |
| MYB51 | Myb domain protein 51 |
| AT1G20130 | Anther-specific proline-rich protein APG (534 aa) |
| SRO2 | Similar to RCD one 2 |
| GH3.17 | Indole-3-acetic acid-amido synthetase GH3.17 |
| NIP3;1 | Aquaporin NIP3-1 |
| AOX1D | Alternative oxidase 3 |
| NAC13 | NAC domain protein 13 |
| BZIP60 | bZIP transcription factor 60 |
| AT1G43910 | P-loop containing nucleoside triphosphate hydrolase-like protein (475 aa) |
| CYCA3;2 | cyclin-A3-2 (372 aa) |
| NUC-L1 | Nucleolin |
| AT1G49050 | Aspartyl protease |
| GSTU16 | Glutathione S-transferase TAU 16 |
| NAC025 | NAC domain containing protein 25 |
| LACS3 | Long-chain acyl-CoA synthetase |
| AT1G66100 | Thionin |
| CMPG1 | CYS, MET, PRO, and GLY protein 1 |
| CCOAMT | caffeoyl-CoA 3-O-methyltransferase |
| AT1G69840 | Hypersensitive-induced response protein 2 (286 aa) |
| GSTU12 | Glutathione S-transferase TAU 12 |
| SRO3 | SRO3-like protein |
| HTH | HOTHEAD |
| CYP98A8 | P-coumarate 3-hydroxylase |
| GSTU10 | Glutathione S-transferase TAU 10 |
| PR5 | Pathogenesis-related gene 5 |
| ATA27 | Beta-glucosidase 20 (535 aa) |
| WAKL10 | WALL ASSOCIATED KINASE (WAK)-LIKE 10 |
| SOT12 | Sulphotransferase 12 |
| DTX1 | Detoxification 1 |
| AT2G04100 | MATE efflux family protein (483 aa) |
| XYP2 | Xylogen protein 2 (169 aa) |
| UGT73B5 | UDP-glucosyl transferase 73B5 |
| CPK16 | Calcium-dependent protein kinase 16 |
| AT2G18193 | P-loop containing nucleoside triphosphate hydrolase-like protein (495 aa) |
| SHT | Spermidine hydroxycinnamoyl transferase |
| AT2G23110 | Late embryogenesis abundant protein, group 6 (92 aa) |
| AT2G25150 | HXXXD-type acyl-transferase-like protein |
| GSTU4 | Glutathione S-transferase |
| C4H | Cinnamate-4-hydroxylase |
| CYS2 | PHYTOCYSTATIN 2 |
| AT2G35615 | Aspartyl protease-like protein (447 aa) |
| AT2G36770 | UDP-glucosyl transferase 73C (496 aa) |
| AT2G36780 | UDP-glucosyl transferase 73C (496 aa) |
| DOGT1 | Don-glucosyltransferase 1 |
| AT2G40360 | transducin/WD-40 repeat-containing protein |
| RHC1A | RING-H2 finger C1A (328 aa) |
| SSL2 | Strictosidine synthase-like 2 (376 aa) |
| AT2G41475 | Embryo-specific protein 3, (ATS3) (179 aa) |
| FBH4 | FLOWERING BHLH 4 (359 aa) |
| COR15B | Cold regulated 15b |
| MBF1A | Multiprotein bridging factor 1A |
| AT2G42990 | GDSL esterase/lipase (350 aa) |
| 5PTase12 | Inositol-polyphosphate 5-phosphatase 12 |
| ARL | ARGOS-like |
| MAP2A | Methionine aminopeptidase 2A |
| ERF071 | Ethylene response factor 71 |
| AT2G47890 | Zinc finger protein CONSTANS-LIKE 13 (332 aa) |
| LBD20 | LOB domain-containing protein 20 (273 aa) |
| NIP7;1 | Putative aquaporin NIP7-1 |
| AT3G08630 | Uncharacterized protein |
| ABCC3 | ATP-binding cassette C3 |
| AT3G13600 | Calmodulin-binding family protein |
| RHD3 | ROOT HAIR DEFECTIVE 3 |
| EIF4A1 | Translational initiation factor 4A-1 (415 aa) |
| DJ1A | DJ-1 homolog A |
| ATA20 | Anther 20 (416 aa) |
| EBP | Ethylene-responsive element binding protein |
| ASPG1 | Aspartyl protease family protein |
| NUC-L2 | Nucleolin |
| ZF2 | Zinc-finger protein 2 |
| GRP5 | Glycine-rich protein 5 (174 aa) |
| PLT1 | PLETHORA 1 |
| AT3G21910 | Domain of unknown function (DUF26) (278 aa) |
| AT3G21920 | Domain of unknown function (DUF26) (278 aa) |
| AOX1A | Alternative oxidase 1A |
| CYP71B23 | Cytochrome P450 71B23 (501 aa) |
| TSO2 | TSO MEANING ’UGLY’ IN CHINESE 2 |
| CYP81D11 | Cytochrome P450 CYP81D11 |
| ATA1 | TAPETUM 1 |
| AT3G46080 | C2H2-type zinc finger protein |
| DMS3 | DEFECTIVE IN MERISTEM SILENCING 3 |
| CML41 | Calmodulin-like 41 |
| LTP12 | Lipid transfer protein 12 |
| KCS15 | 3-ketoacyl-CoA synthase 15 (451 aa) |
| SIB1 | Sigma factor binding protein 1 |
| TT12 | TRANSPARENT TESTA 12 |
| BGLU27 | Beta glucosidase 27 (540 aa) |
| CRF6 | Cytokinin response factor 6 |
| AT3G62120 | prolyl-tRNA synthetase |
| AT4G00040 | Chalcone synthase 2-like protein |
| ATHSP22.0 | Heat shock protein 22 (195 aa) |
| AT4G10490 | Oxidoreductase, 2OG-Fe(II) oxygenase family protein |
| MEE48 | Maternal effect embryo arrest 48 |
| MO1 | Monooxygenase 1 (422 aa) |
| HDG4 | Homeodomain GLABROUS 4 |
| HSFA4A | Heat shock transcription factor A4A |
| QRT3 | QUARTET 3 |
| GA2OX8 | Gibberellin 2-oxidase 8 |
| ATG8A | AUTOPHAGY 8A |
| DTX35 | Detoxifying efflux carrier 35 |
| At4g26970 | Aconitase 2 |
| ADR1-L1 | ADR1-like 1 |
| AT4G33355 | Non-specific lipid-transfer protein 11 |
| UGT73B1 | UDP-glucosyl transferase 73B1 |
| LHT7 | LYS/HIS transporter 7 |
| PLP4 | PATATIN-like protein 4 |
| ACS8 | Encodes an auxin inducible ACC synthase |
| AT4G37900 | Uncharacterized protein |
| UGT76C1 | UDP-glucosyl transferase 76C1 |
| PGIP1 | Polygalacturonase inhibitor 1 |
| EMB1873 | Agmatine deiminase |
| ATAF2 | Protein ATAF2 (283 aa) |
| PBS3 | AVRPPHB SUSCEPTIBLE 3 |
| TT4 | Chalcone synthase |
| AT5G16990 | 2-alkenal reductase |
| DAR4 | DA1-related protein 4 |
| CYP86B1 | Cytochrome P450 86B1 |
| ATXR6 | ARABIDOPSIS TRITHORAX-RELATED PROTEIN 6 |
| WRKY50 | WRKY DNA-binding protein 50 |
| GORK | Potassium channel GORK |
| NAC6 | NAC domain containing protein 6 |
| At5g49480 | Ca2+-binding protein 1 (160 aa) |
| AT5G49680 | Putative cell expansion protein |
| AT5G51440 | Heat shock protein 23.5 (210 aa) |
| AT5G55150 | Uncharacterized protein (360 aa) |
| GEX1 | Gamete expressed protein 1 |
| PFK7 | Phosphofructokinase 7 |
| FIP1[V] | FIP1 [V]-like protein |
| RHL41 | RESPONSIVE TO HIGH LIGHT 41 |
| AT5G59845 | Gibberellin-regulated protein |
| cPT9 | Cis-prenyltransferase 9 |
| GSTU9 | Glutathione S-transferase tau 9 |
| AT5G64460 | Phosphoglycerate mutase-like protein |
| PSBO1 | PS II oxygen-evolving complex 1 |
| PB | ATP synthase subunit beta |
| RBCL | Ribulose-bisphosphate carboxylases |
| PETA | Photosynthetic electron transfer A |
| PSBB | Photosystem II reaction center protein B |
| ORF25 | Hydrogen ion transporting ATP synthases, rotational mechanism |
